# Biophysical Assessment of Single Cell Cytotoxicity: Diesel Exhaust Particle-Treated Human Aortic Endothelial Cells

**DOI:** 10.1371/journal.pone.0036885

**Published:** 2012-05-25

**Authors:** Yangzhe Wu, Tian Yu, Timothy A. Gilbertson, Anhong Zhou, Hao Xu, Kytai Truong Nguyen

**Affiliations:** 1 Department of Biological Engineering, Utah State University, Logan, Utah, United States of America; 2 Department of Biology, Utah State University, Logan, Utah, United States of America; 3 Department of Bioengineering, The University of Texas at Arlington, Arlington, Texas, United States of America; Emory University/Georgia Insititute of Technology, United States of America

## Abstract

Exposure to diesel exhaust particles (DEPs), a major source of traffic-related air pollution, has become a serious health concern due to its adverse influences on human health including cardiovascular and respiratory disorders. To elucidate the relationship between biophysical properties (cell topography, cytoskeleton organizations, and cell mechanics) and functions of endothelial cells exposed to DEPs, atomic force microscope (AFM) was applied to analyze the toxic effects of DEPs on a model cell line from human aortic endothelial cells (HAECs). Fluorescence microscopy and flow cytometry were also applied to further explore DEP-induced cytotoxicity in HAECs. Results revealed that DEPs could negatively impair cell viability and alter membrane nanostructures and cytoskeleton components in a dosage- and a time-dependent manner; and analyses suggested that DEPs-induced hyperpolarization in HAECs appeared in a time-dependent manner, implying DEP treatment would lead to vasodilation, which could be supported by down-regulation of cell biophysical properties (e.g., cell elasticity). These findings are consistent with the conclusion that DEP exposure triggers important biochemical and biophysical changes that would negatively impact the pathological development of cardiovascular diseases. For example, DEP intervention would be one cause of vasodilation, which will expand understanding of biophysical aspects associated with DEP cytotoxicity in HAECs.

## Introduction

Diesel exhaust particle (DEP) exposure-related urban air pollution contributes to morbidity and mortality through an increase in the incidence of cancer and other health problems [1]–[3]. DEPs are directly emitted from diesel-powered engines and serve as a major source of traffic-related air pollution, and it is mainly composed of a carbonaceous core, upon which organic compounds, nitrate and many other compounds are adsorbed [4]. Previous reports indicated that DEPs can not only invade either the upper or lower respiratory tracts and the alveolar region depending on the particle size (larger or smaller than 2.5 µm) [4], but also result in severe impairments in physiological function of the cardiovascular system [5]–[7]. However, it remains controversial concerning how DEPs are transported to interact with endothelium. A popular view believes that a fraction of DEPs (e.g. less than 100 nm in size) could penetrate deeply into the respiratory track after intratracheal inhalation and translocate from the lungs into the circulation, which could lead directly to interact with the endothelium [6], [8]. While it generally accepted that DEPs can potentially induce dysfunction in cardiovascular system, it is not yet clear if DEPs are capable of inducing biophysical alterations of vascular cells, especially vascular endothelial cells (VECs), at the single cell level. Therefore, it is of interest to evaluate these interactions, which can help further elucidate the mechanisms underlying the ability of DEPs to functionally impair endothelial cells. Atomic force microscopy (AFM), a powerful force (nN/pN)-sensitive technique, had been successfully applied in single cell studies. This technology can provide information on cell topography, membrane nanostructures and mechanics (e.g. adhesion force, elasticity) of mammalian cells [9]–[11] at a nanoscale resolution under physiological or near-physiological conditions [9], [12]. Therefore, AFM should allow researchers to better understand biophysical responses of mammalian cells in the presence of DEPs-related air pollution and etiopathology. Previous applications of AFM in endothelial cell studies include cell mechanical measurements [13]–[15], recognition imaging of surface receptors [16]–[18], and also cell topographical features [19], showed the feasibility and utility of AFM to qualitatively and quantitatively detect cell structures, mechanics and functions of living endothelial cells [20]. In the present work, to bring new insights into the toxic effects of DEPs on VECs and eventually into DEP-related dysfunction of the cardiovascular system, AFM, fluorescence microscopy, and flow cytometry were applied to analyze the toxic effects of DEPs on human aortic endothelial cells (HAECs) at single cell level.

## Materials and Methods

### HAEC culturing

In our experiments, Human Aortic Endothelial Cells (HAECs; Cascade Biologics, USA) were selected as a cell model for our research. The cells were cultured in complete media consisting of culture medium 199 (M199) supplemented with 20% FBS, 5% low serum growth supplement (LSGS), ∼20 ng/ml endothelial growth factor (EGF), and 1% penicillin–streptomycin (all from Invitrogen). Cells were incubated in a humid environment at 37°C and 5% CO_2_. Upon 80–90% confluency, the cells were either passaged or used for cell viability tests; for AFM related experiments, HAECs were used when confluency reached ∼50%. In the present study, cells used were within 3∼5 generations of the initial passage.

### Diesel exhaust particles

Diesel exhaust particles (DEPs) were generously provided by Dr. M. Ian Gilmour of the National Health and Environmental Effects Research Laboratory, U.S. Environmental Protection Agency (Research Triangle Park, NC). According to our primary evaluation, DEP size ranges from approximately tens of nanometers to as large as 2 µm in M199 culture medium (Information S1). Even though DEPs are generally hydrophobic in nature, we found that they are relatively easily suspended in complete cell growth medium (M199) using a vortex-shaking method followed by sonication for 30 min at room temperature. The stock solution of DEP suspension was added directly into culture medium to produce appropriate working concentrations. To evaluate effects of DEPs (as a function of concentration and treatment time) on cells, three working concentrations of DEPs were used: 10 µg/ml (which is approximately to be 3.12 µg/cm^2^), 50 µg/ml, and 100 µg/ml; and four exposure times were studied: 4, 8, 24, and 48 hours. We empirically determined that to induce visible alterations for AFM observations or measurements and fluorescence imaging during *short-term exposure* required a minimum concentration of 10 µg/ml DEPs, even though this concentration may be higher than that commonly found in long-term environmental exposures.

### Cell viability assessment

To test cytotoxicity of DEPs, the HAEC viability was analyzed using LIVE/DEAD Viability/Cytotoxicity Assay Kit (Invitrogen) according to the manufacturer's instruction. Briefly, (1) HAECs cultured in culture flask were harvested and then suspended in fresh growth media, and 2 mL of cell suspension was seeded into poly-D-lysine coated glass-bottom dishes (MatTek Cop. USA) and further cultured for 3 days, following by DEP treatment; (2) cells were then washed two times with serum-free essential media (M199) prior to addition of fluorescent reagents; (3) 200 µl of mixed solution of 2 µM Calcein AM and 4 µM ethidium homodimer-1 (EthD-1) (both from Invitrogen) was added directly to cells, and incubated cells for 30 mins at room temperature; (4) fluorescent images were acquired using a fluorescence microscope with DP30BW CCD camera (Olympus IX71) to analyze the relative proportion of live/dead cells. Here, a 10× objective was used to observe fluorescence. Calcein AM is well retained within living cells producing green fluorescence; however, EthD-1 enters cells with damaged membrane and binds to nucleic acids, thereby producing a red fluorescence in dead or membrane-damaged cells. Therefore, the live/dead cells were differentiated visually.

### Measurements of cell surface ultrastructures and cell mechanics

To perform the AFM experiments, cells were seeded on poly-D-lysine-coated Petri dishes (MatTek) at a density of 1×10^5^ cells per 2 mL of media and then cultured for 4 days. Cells treated with or without DEPs were then measured by AFM.

For AFM measurements, two approaches of sample preparation were adopted to measure cell mechanics or perform cell imaging. **1**) The *in situ* approach was used to measure the mechanical properties such as adhesion force and Young's modulus; that is, cells grown on poly-D-lysine-coated Petri dishes were directly transferred onto AFM scanner stage for measurements after cells were washed two times with fresh culture medium (without any additional pretreatment), and measurements were conducted in whole culture medium (M199) at room temperature; the acquired data thus reflected the physiological status of the observed living cells. **2**) To visualize topography and membrane nanostructures, the cells were pretreated by fixing with 1% glutaraldehyde plus 1% paraformaldehyde dissolved in 1× Ca^2+/^Mg^2+^-free phosphate buffered saline for 5 min; followed by gentle rinsing using PBS. The Petri dish containing the cells immersed in PBS was then transferred onto the AFM scanner stage for imaging. While the observed results only reflected the quasi-physiological state of cells, the image resolution and quality were vastly improved.

The contact mode Picoplus AFM controlled by software PicoScan 5.3 (Agilent Technologies, USA) was used to perform measurements in PBS (0.01 M, pH 7.4) or directly in cell culture medium. During AFM imaging, height and deflection mode images were acquired simultaneously. Because the deflection mode image usually can provide greater fine structure details than height mode images, especially for larger mammalian cells, the deflection mode image was used throughout these studies; however, experimental analyses like cell surface roughness were conducted based on height mode images. The spring constant of the cantilever used in the experiments was 0.06 N/m (Vecco). The length of tip with pyramid shape is ∼3 µm, and the curvature radius of the Si_3_N_4_ tip is approximately 10 nm. Also, the approach/retract velocity applied throughout the experiments of deflection (nm) vs. distance (nm) curve acquirement was 6 µm/s. The values for the adhesion force (the detachment force between bare AFM tip and cell surface in the process of AFM cantilever retracting, which reflects alteration of cell membrane adhesion behavior/property or denaturation of membrane surface adhesion molecules) were extracted from force curves via the Scanning Probe Image Processor (SPIP) software (Image Metrology, Denmark). By applying the AFM tip to the cell surface, the elasticity modulus of cells can be evaluated based on the slope of compliance portion of the deflection-distance curves. To analyze alterations in cell elasticity, Young's modulus was calculated according to the formula [9], [21]:
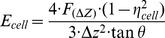
(1)Where, ***E_cell_***, cellular Young's modulus; *F*, loading force; ***η_cell_***, Poisson ratio (assuming 0.5); **Δz**, indentation; *θ* is the tip half opening angle that equals to 36°.

With this formula, we calculated the Young's modulus based on hundreds of deflection-distance curves acquired for each group.

Furthermore, to assess statistically significant differences in biophysical properties including adhesion force and cellular elasticity between control group and experimental groups, the data were reported as mean±SE (standard error of the mean), and the statistical difference was analyzed by a Student's t-test or one-way ANOVA. The data used to graph histogram or maps were measured from the entire cell body, and the histograms were drawn using OriginPro 7.5 (OriginLab Corp., USA). The mapping of adhesion force or cellular spring constant was graphed with a 256 color map created by Matlab program version R2009a (MathWorks, Inc.) according to previously described methods [9], [22].

### Cell staining for observation using coupled atomic force/fluorescence microscope

The coupled atomic force/fluorescence (AF/FL) microscope system consisted of PicoPlus AFM and inverted fluorescence microscope (Olympus IX71) with a specially designed stage (Agilent Technologies). The imaging conditions/parameters for AFM are similar to those mentioned above; for fluorescence microscope, 60× or 100× oil immersed objective lens (OIL) was used to acquire bright-field and fluorescence images.

To perform AF/FL imaging, cell suspensions of HAECs were seeded into poly-D-lysine-coated glass-bottom Petri dishes and further cultured in the medium for 4 days, then followed by DEP treatments at three concentrations and four exposure times. Cell samples were stained with fluorescent phallotoxin and DAPI dihydrochloride by referring to the Invitrogen protocols. For staining, cells were washed twice PBS, fixed in 3.7% formaldehyde solution in PBS for 10 min at room temperature (RT); treated with 0.1% Triton X-100 in PBS for 3 min at RT; incubated with 1% BSA for 60 min at RT, followed by incubation with 200 µl of fluorescent phallotoxin (working concentration of 160 nM) for 30 min at RT in the dark; then incubated with 200 µl of DAPI (300 nM) for 5 min at RT in the dark; and finally, the stained cell samples were immersed in PBS and stored at 4°C refrigerator before use. Cells samples were washed twice using PBS between each of steps. Prepared cell samples were then observed by the coupled AF/FL microscope in PBS.

### Flow cytometry

Flow cytometry measurement was performed with BD FACSAria II (BD Biosciences, San Jose, CA), and data analysis was conducted by FACSDiva version 6.1.3 (BD Biosciences). After several attempts, we found that higher concentrations such as 50 µg/ml or 100 µg/ml of DEP-treated cells were not suitable for flow cytometry analysis because of the interference of DEPs and significant decreases in cell number. Therefore, only 10 µg/ml of DEPs was used for subsequent flow cytometry assessments.

#### Plasma membrane potential

The dye *bis*-(1,3-Dibarbituric acid)-trimethine oxanol (DiBAC4(3)) (Enzo Life Sciences, Ex/Em: 493/516 nm) was applied to detect the alterations in plasma membrane potential of HAECs in the absence and presence of 10 µg/ml DEPs. DiBAC4(3) serves as an indicator for the changes in membrane potential. An increase in fluorescence intensity indicates depolarization of cells, while a decrease in fluorescence intensity is indicative of cell hyperpolarization. The harvested DEP-treated cells were incubated in 5 µM dye-PBS solution in 37°C incubator with 5% CO_2_ for 30 min. Then, stained cells were tested using flow cytometry immediately without washing.

## Results

### Effects of DEPs on cell viability

**Figure 1:**
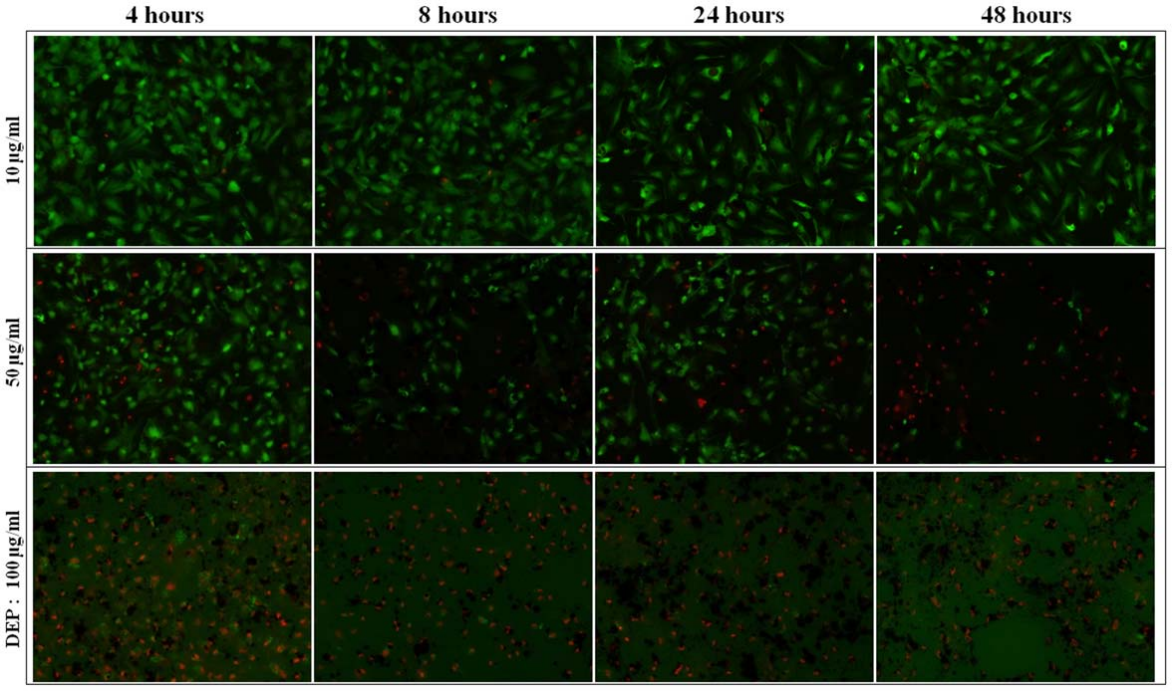
Representative fluorescence images of cell viability evaluation. HAECs cells were exposed to DEPs with three different concentrations: 10 µg/ml (row 1), 50 µg/ml (row 2), 100 µg/ml (row 3), and four treatment durations: 4 hours (column 1), 8 hours (column 2), 24 hours (column 3), and 48 hours (column 4), respectively. Cells are stained with Invitrogen LIVE/DEAD Viability/Cytotoxicity Assay Kit. Green fluorescence presents live cells, whereas red fluorescence shows dead or membrane-damaged cells. All images were obtained with 10× lens. These fluorescence images together revealed that DEPs impaired cell viability in a dosage- and a time-dependent manner. And the corresponding bright-field picture of each fluorescent image is shown as Information S5, from which DEPs can be clearly seen.

To assess cytotoxicity of DEPs, cell viability was evaluated first using fluorescence microscopy, and representative results are shown in Figure 1 and Information S2. Fluorescence images in rows 1–3 show three groups of cell samples treated by different concentrations of DEPs, 10 µg/ml, 50 µg/ml, and 100 µg/ml, respectively. The four DEP-cell exposure time points (4, 8, 24, 48 hours) for each group are arranged as columns (1–4), respectively. As mentioned in “*Materials and Methods*” section, green fluorescence is indicative of living cells, whereas red fluorescence shows dead or membrane-damaged cells. Fluorescence images indicate that 10 µg/ml of DEP treatment did not exert significantly effects on cell viability even after 48 hours of exposure (row 1 of Fig. 1), and there are only very a few dead or damaged cells (red color) for all four time points, however, increasing DEP concentration lead to a visible reduction in cell viability (rows 2 and 3 of Fig. 1). For example, for 50 µg/ml of DEP treatment, results showed that the percentage of dead or membrane-damaged cells clearly elevated for all four time points compared with the 10 µg/ml group, and that the majority of cells were dead or membrane-damaged after treating for 48 hours. For higher concentrations of DEPs (100 µg/ml), it was found that red fluorescence is predominant for all four time points, suggesting that the majority of cells were already dead or membrane-damaged even at the first 4 hours of exposure. These fluorescence images together revealed that DEPs impaired cell viability in a dosage- and a time-dependent manner.

### DEPs-induced ultrastructural changes

**Figure 2:**
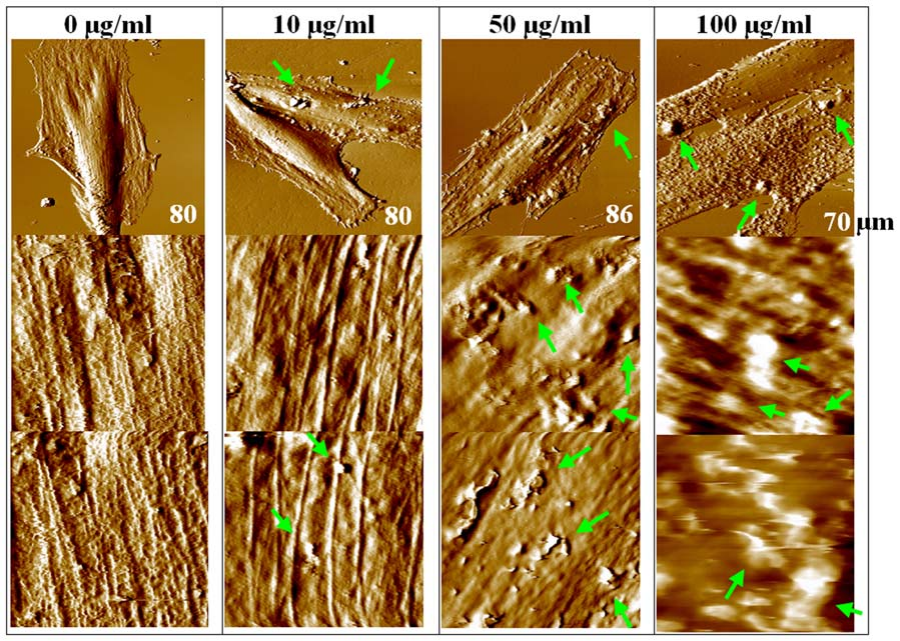
Representative AFM deflection mode images of fixed HAECs obtained in PBS. Column 1, images of untreated cells (0 µg/ml group); columns 2–4, images of cells treated with different concentrations of DEPs for 4 hours. Row I, image of single cells, whose scanning size is marked on respective image; rows II and III, images of membrane surface ultrastructures, whose scanning size is 10 µm×10 µm. Particles (representatively shown by green arrows) on cells are DEPs. This group of images indicated that when DEP concentration increased from 0 µg/ml to 100 µg/ml, cytoskeletal structures became gradually degraded, suggesting that cell membrane damage appeared in a dosage-dependent manner. And poor resolution of ultra-structures of 100 µg/ml DEP treated cells is mainly resulted from influences of the large amount of DEPs on membrane surface.

**Figure 3:**
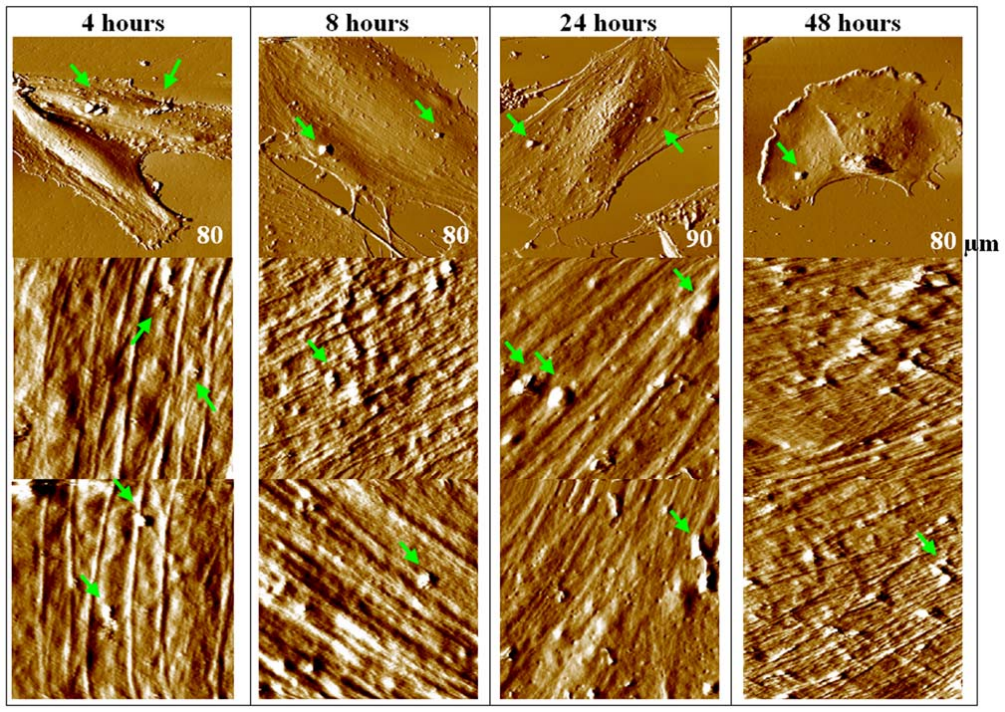
Representative AFM deflection mode images of HAECs treated with 10 µg/ml DEPs for different time (cells were fixed prior to observe in PBS). Column 1 shows images of cells treated with DEPs for 4 hours; column 2, 8 hours; column 3, 24 hours; column 4, 48 hours. Row 1 shows deflection mode images of single cells; rows 2 and 3 show images of membrane surface ultrastructures. Particles on cells representatively indicated by green arrows are DEP. Scanning size of row 1 is marked on respective image; and that of row 2 and row 3 (ultrastructures): 10 µm×10 µm. This group of images indicated that when treatment time increased from 4 hours to 48 hours, cell membrane damage appeared in a time-dependent manner.

To achieve visualization of topographical and membrane nanostructures of HAECs in the context of DEP treatment, AFM was applied to perform imaging in PBS solution. Cell samples were treated with different concentrations (0, 10, 50, and 100 µg/ml) of DEPs for various exposure time including 4, 8, 24, and 48 hours, and the results are shown in Figures 2, 3 and Information S3. Here, only deflection mode images were used to display AFM observations, because this mode can more readily reveal fine structural details than the height mode. Figure 2 shows representative images of HAECs treated without (column 1) or with three concentrations of DEPs for 4 hours (columns 2–4), and Figure 3 exhibits results of cells treated with 10 µg/ml DEPs for 4 different exposure time. Images of untreated cells exhibit apparent lamellipodia around cells with height of 40∼700 nm, and ultrastructural images clearly reveal linearly aligned cytoskeletons, which are interconnected to each other by filamentous structures, yielding a mesh-like array. When DEP concentrations were increased from 10 µg/ml to 100 µg/ml, membrane ultrastructures were altered visibly (Fig. 2); for example, cytoskeletal structures that are easily seen for untreated cells and 10 µg/ml DEPs treated cells became progressively degraded and more and more obscure (Figs. 2, 3; Information S3). Together these results suggested that DEP-induced cell damage appeared in a dosage-dependent manner. Further, AFM images also suggested that cell membrane damage appeared in a time-dependent manner. For example, for the 10 µg/ml DEP-treated cell group (Fig. 3), when treatment time increased from 4 hours to 48 hours, cytoskeletal structures became gradually degraded. Additionally, there are many punctate particles, which were readily seen for all DEPs treated groups (representatively pointed by green arrows), adhered to the cell membrane surface. These particles appear to be the DEPs according to further observations of AF/FL microscope (Information S4) and bright-field images of fluorescence microscopy (Information S5).

### Evaluation of cell mechanics of DEPs treated HAECs

**Figure 4:**
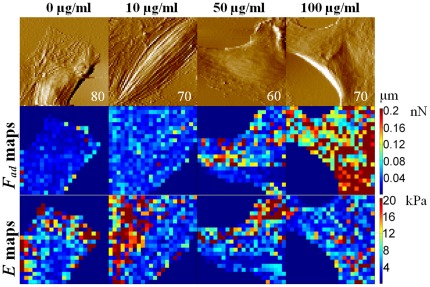
Maps of cell mechanics including adhesion force (*F*_ad_, nN) and Young's modulus (*E*, kPa) of live HAECs, which were measured on single cells in culture medium. These maps are only to exhibit heterogeneous property in cell mechanics and do not reflect alteration tendency of *F*_ad_ or *E* because of individuality of cell-cell. Column 1, untreated (0 µg/ml) cells; columns 2–4, images of cells treated with different concentrations of DEPs for 4 hours. Row 1 shows AFM deflection mode images; rows 2 and 3 are their corresponding maps of adhesion force and Young's modulus. The color bars showing at the right of maps display the value scale of *F*_ad_ and *E*. The scanning size of AFM images is marked on each image of row 1.

After observing ultrastructural changes in the cell membrane, especially the cytoskeletal changes, it was of interest to investigate the biomechanical properties of HAECs in the absence and presence of DEPs, which would offer new insights into assessments of DEP cytotoxicity. Therefore, AFM was used to quantify mechanical changes (including membrane adhesion force and cell elasticity) of HAECs in culture medium, and results measured on single cells are shown in Figure 4 and Information S3. The maps of adhesion force (*F*_ad_, nN) and Young's modulus (*E*, kPa) exhibited heterogeneous distribution of adhesion behavior and elasticity over the whole cell body, for example, the region of cell nucleus possesses a lower elasticity comparing to region of cytoplasm, and this feature commonly appeared in the Young's modulus maps of all experimental groups (Fig. 4; Information S3).

**Figure 5:**
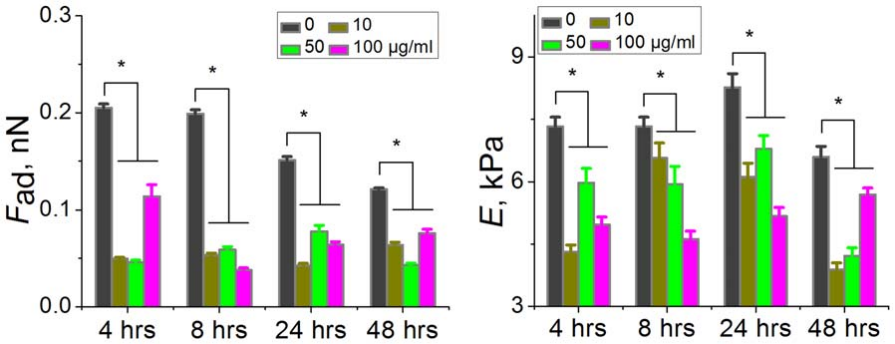
Statistical analysis of adhesion force (*F*_ad_, nN) and Young's Modulus (*E*, kPa) of live HAECs. Decreases of adhesion force implicates depression of cell membrane adhesion behavior in the presence of DEP, and down-regulation of Young's modulus suggests cells become softer in the context of cytoskeleton losing induced by DEP treatment. The data of histograms were obtained from multiple cells (N_cell_ = 12 for each group, and 26 datum points on each cell). Error bar: standard error (SE). (*, P<0.01).

Furthermore, a statistical analysis of alterations of adhesion force (*F*) and cell elasticity (Young's modulus, *E*) obtained from multiple cells was conducted, and their respective histograms are shown in Figure 5. Both histograms of adhesion force and Young's modulus clearly indicated significant decreases occurred after cells were treated with DEPs. However, both of these two parameters did not show consistent dosage- or time- dependent alteration tendency.

### Cytoskeleton visualizations using coupled AF/FL microscope

**Figure 6:**
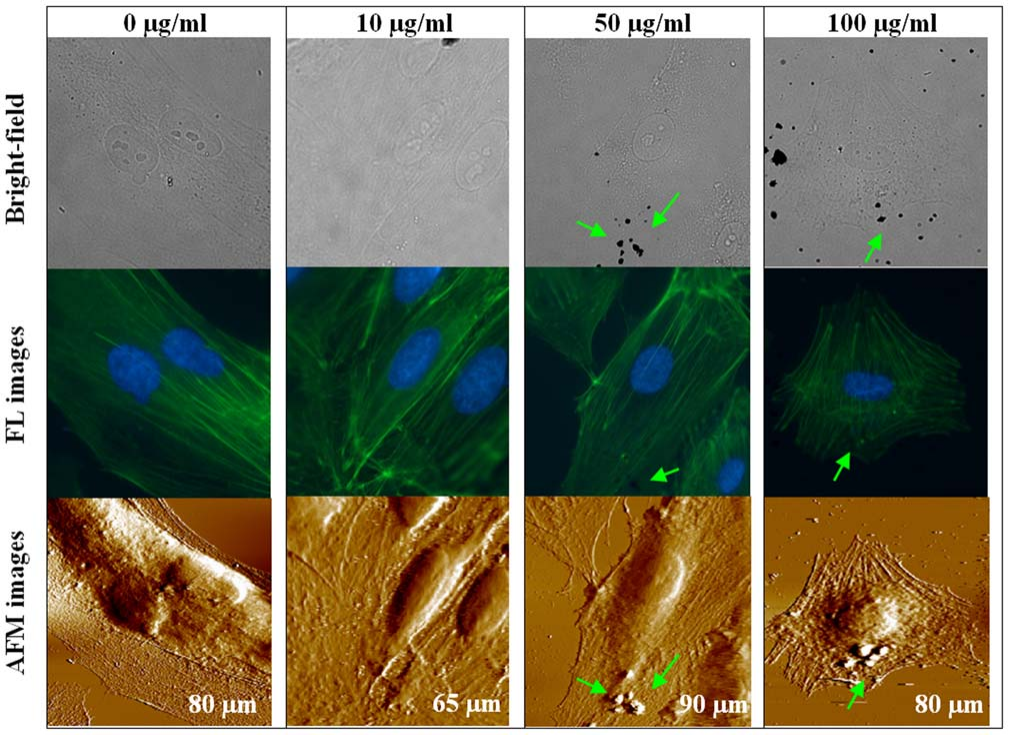
Representative observations of fixed HAECs using the coupled AF/FL microscope in PBS. Column 1 shows images of untreated (0 µg/ml) group; columns 2–4 are images of cells treated with different concentrations of DEPs for 4 hours. Row 1 shows bright-field images, row 2 is fluorescence images, and row 3 exhibits AFM images of the same cells. Optical images were obtained by the 60× OIL (or 100× OIL for 10 µg/ml group); scanning size of AFM images is marked on the respective images. The black dots representatively pointing by green arrows are DEPs attached on cell membrane.

**Figure 7:**
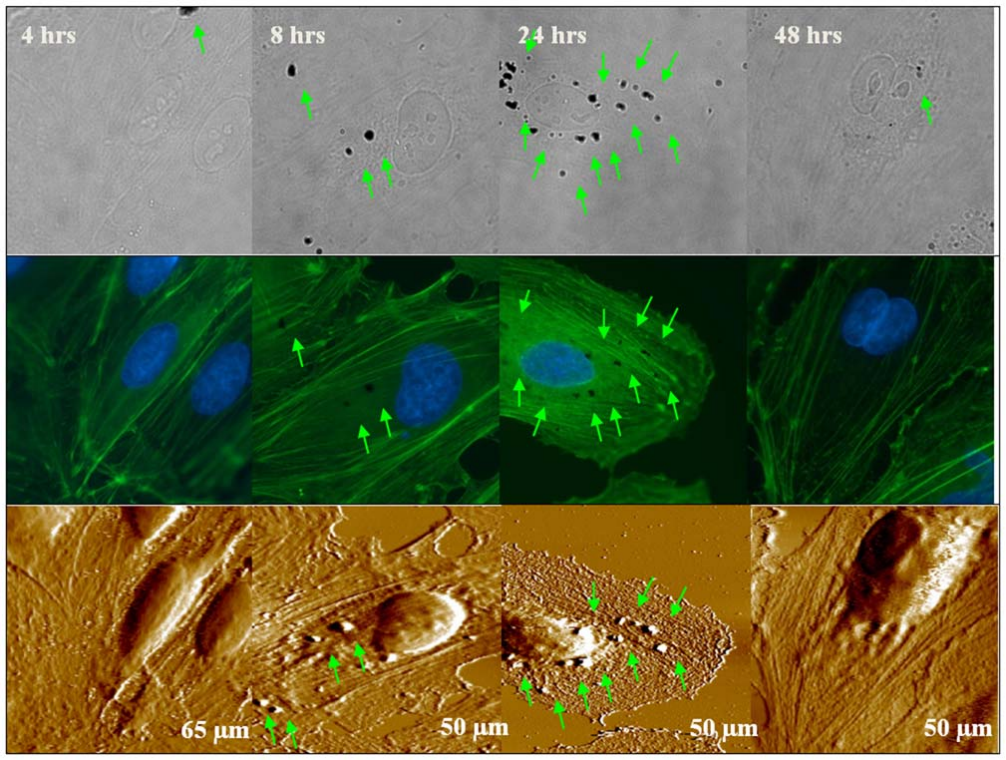
Representative images of DEP (10 µg/ml) -treated HAECs (fixed cells) in PBS obtained by the coupled AF/FL microscope. The panel arrangement is similar to Figure 6. Optical images were obtained using a 100× oil objective. The exposure time of DEPs is marked on respective optical images. It is seen that fluorescence intensity slightly decreased, although cytoskeletal structures can be seen after 48 hours of treatment. Interestingly, cellular mitosis was still progressing and a dividing cell nucleus can be seen (column 4), implying this low dosage did not completely inhibit cell activities, which coincided with assessment of cell viability. Additionally, DEPs attached on cell membrane surface are seen for all four experimental groups, as indicated by green arrows.

To further detect potential alterations of cytoskeletal structures of HAECs treated with three concentrations of DEPs for different exposure times, a coupled atomic force/fluorescence (AF/FL) microscope was applied to simultaneously perform fluorescence and topography imaging, as shown in Figures 6, 7 and Information S4. For untreated cells, cellular cytoskeletal structures arranged in parallel can be readily seen from the fluorescence and topography images (column 1 of Fig. 6).

Furthermore, images obtained by the AF/FL microscope visualized that cellular cytoskeletons were impaired by DEPs in a dosage- and a time-dependent manner (Figs. 6, 7; Information S4). For example, for 10 µg/ml DEPs treated cells (Fig. 7), observations show that fluorescence intensity slightly decreased although cytoskeletal structures can be seen after 48 hours of treatment. However, when concentrations of DEPs were elevated to 50 µg/ml or 100 µg/ml, green fluorescence intensity (depicting cytoskeleton contents) dimmed more rapidly, which is dependent on both exposure time and DEP concentration, suggesting that a higher dosage of DEPs leads to gradual down-regulation of cytoskeleton components. Additionally, after long-term treatment (e.g. 24 or 48 hours) of high concentration of DEPs, cell shape and cell architecture especially lamellipodia became obviously contracted (Information S4), and cell shape was hardly recognizeable in the fluorescence images.

### Plasma membrane potential analysis

**Figure 8:**
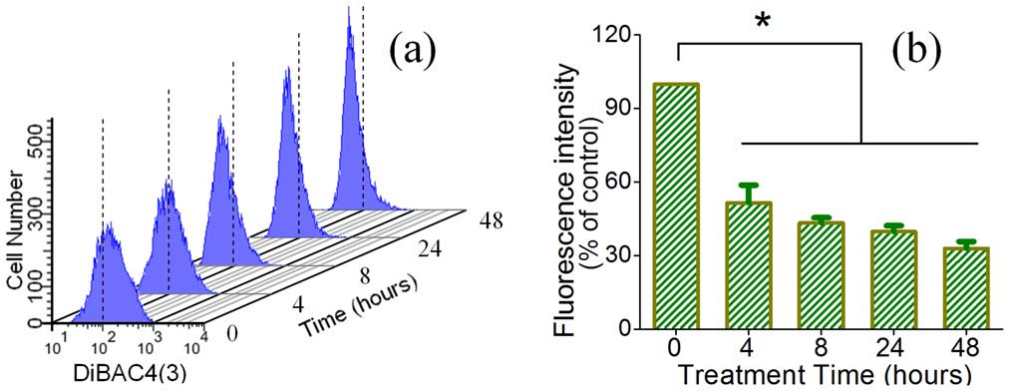
Changes of plasma membrane potential of live HAECs treated with or without 10 µg/ml DEPs assayed by flow cytometry. (a) Representative graphs show that DEP exposure induced a significant decrease in fluorescence intensity of DiBAC4(3). (b) Histograms of changes in fluorescence intensity. Fluorescence intensity of experimental groups is presented as a percentage of control (0 hour) (that of control group was normalized as 1). Results represent the means of three separate experiments, and error bars represent the standard error of the mean. All experimental groups significantly differs from control group (*p<0.01).

To detect effects of DEP exposure on cell membrane function, alterations of plasma membrane potential (PMP) of HAECs in the absence and presence of DEPs were also evaluated by flow cytometry, and results are shown in Figure 8. Figure 8A exhibits representative graphs of fluorescence intensity of untreated cells (0 hours) and DEP-treated cells (4∼48 hours), and Figure 8B shows histograms of statistical analyses based on three independent experiments, showing significant time-dependence decreases in PMP after treating with DEPs (p<0.01), which suggested dysfunction or damage of the plasma membrane.

## Discussion

Cytotoxicity of diesel exhaust particles (DEPs) evokes increasing concerns with adverse effects on human health, especially on the cardiovascular system. Previous approaches to study cytotoxicity associated with DEP exposure were based mainly on traditional molecular biology, cytotoxicity or pathology methods. However, none of these traditional methods provides any information of cell biophysical properties, including cell membrane nanostructures, membrane integrity, and cell biomechanics; notably lacking analyses of these properties at the single cell level. Thus, investigations of such biophysical information using nanotechnologies will further elucidate DEP-induced cytotoxicity at the single cell level. Inspired by this motivation, in the present work, atomic force microscopy was utilized to detect biophysical and biochemical changes at the single cell level for our model cell (human aortic endothelial cells, HAECs) in the absence and presence of DEPs; and a coupled microscope system by combining atomic force microscopy and fluorescence microscopy (AF/FL) was used to visualize possible re-arrangement and even destruction of cytoskeletal elements induced by DEPs.

Fluorescence results suggested that high concentration and long-term exposure of DEPs could destroy cell bodies itself while inducing cell death or cell membrane damage, and that DEP-induced cell death or membrane damage appeared in a dosage-dependent and a time-dependent manner. For example, AFM observations (Fig. 3) indicated that 10 µg/ml DEPs did not obviously change cellular pseudopodia and lamellipodia within 24 hours, whereas, retraction of lamellipodia occurred after 48 hours of treatment, showing that long-term treatment of low DEP concentration could still induce cell topography alteration. These adverse changes became much more severe after cells were treated with higher concentrations of DEPs (≥50 µg/ml). Additionally, alterations of membrane nanostructures revealed that DEP treatment-induced changes of cellular architecture also appeared in a dosage-dependent and time-dependent manner. Such findings implied that cell membrane function would be altered because function depends on structures, and this relationship was supported by experiments using flow cytometer. These results together suggested that VECs are sensitive to DEP intervention, that cytotoxicity of DEPs was externalized as damage of cell architectures like membrane nanostructures, and that AF/FL system could be a promising method for detecting such changes.

Studies suggested that cell mechanics could be a promising “biomarker” for cell phenotypic events associated with changes in cell architecture like topography and membrane nanostructures [9], [10], [23], [24]. Cell mechanics such as elasticity and stiffness play important roles in the regulation of cell structures and functions at the molecular and cellular level [25], [26]. Therefore, investigations of the effects of DEPs on cell mechanics of HAECs at the single-cell/subcellular level could expand understanding the development of cardiovascular disorders associated with particulate matter related urban air pollution. In this work, mechanical maps measured on single endothelial cells revealed that region of cell nucleus possesses a lower elasticity comparing with cytoplasm, indicating heterogeneity in adhesion behavior and elasticity over the whole cell surface (Fig. 4; Information S3). Mechanical heterogeneity is very likely a common feature of cells, which could be supported by our previous works on breast cancer cells [9], lung cancer cells (data not published), bacterial cells [22], and reports from other investigators [14], [27]. Furthermore, statistical analyses of cell mechanics acquired from multiple cells indicated that both adhesion force and cell elasticity were significantly, though non-linearly, down regulated after treating with DEPs, implying cytoarchitectural changes occurred. Decreases in membrane surface adhesion force suggested depression of membrane surface adhesion behavior/property that would implicate denaturation of membrane surface adhesion molecules, whereas, down regulation of cell elasticity was mainly due to decreases in cytoskeleton components (e.g. disaggregation of F-actin), as evidenced by fluorescence results (Figs. 6, 7; Information S5 and S6). Even though previous studies suggested that exposure to particle matters could up-regulate expression of adhesion molecules in human umbilical vein endothelial cells [28], [29], our characterizations of membrane adhesion behaviors of single cells implied that increases of adhesion molecules and decreases of adhesion force could co-occur in the context of DEPs because of possible denaturation of these molecules caused by DEPs. Furthermore, non-linear changes in these two parameters might arise from complex compositions and non-uniform solubility of DEPs in culture medium, and miscellaneous influences of DEPs on cell mechanics, such as adverse effects on cellular architectures [30], [31], or even inverse effects to promote endothelial growth [2].

It is known that cell elasticity mainly originates from the cytoskeleton, especially F-actin. Since DEP treatment induced decreases in cell elasticity, it is meaningful to explore how cytoskeleton organization was altered by DEP intervention. Changes in cell topography and cytoskeleton (Figs. 6, 7; Information S4), obtained using a combined AF/FL setup, revealed that down-regulation in cytoskeletal components (seen as decreases in green fluorescent intensity) appeared in a time- and dosage-dependent manner, indicating a gradual destruction of cytoskeleton organization. Our results presented here are in accordance with a reported observation that cytotoxicity of high concentrations of DEPs is largely due to cytoskeletal dysfunctions [31]. AF/FL analyses clearly revealed that DEP exposure induced disaggregation of the cytoskeleton by the way of actin breakage or degradation, which could eventually result in cell death and detachment from substrate [30]. It is interesting to note here that data from 1000 µg/ml DEP-treated cells were also collected (Information S2, S3, S4). Though concentrations in this range are well in excess of those found environmental exposure, showing such a high concentration of DEP could result in cell death and changes of membrane surface structures and cytoskeleton even 4 hours of exposure; and results also indicated that DEP treatment could gradually impair structure of cell nucleus and eventually result in destruction (Information S4).

Since DEPs could impair cell viability, change cell topography and membrane nanostructures, and induce cytoskeletal reorganizations or destructions, investigation of cell membrane functions can expand our understanding of cytotoxicity of DEPs. Evaluation of plasma membrane potential (PMP) using flow cytometry (Fig. 8) showed a significant decrease in fluorescent intensity as a function of DEP-cell interaction time at the dosage of 10 µg/ml. The dye BiBAC4(3) can enter depolarized cells, and more depolarization in cells will result in more influx of the dye which in turn elevates fluorescence intensity. On the other hand, decreasing in fluorescence intensity of cells caused by extrusion of dye suggests increasing hyperpolarization is occurring [32], [33]. Depolarization is due to change in transmembrane potential or voltage, which is often caused by efflux of cations or influx of anions through their respective channels [33], [34]. When the membrane is compromised (due to toxic DEP), the ability to maintain more negative resting potentials become impaired and the membrane potential is reduced (i.e. more positive or depolarized). Measurements suggested that at low concentration of DEPs (10 µg/ml) could denature membrane structures or conformations and thus interfered or inhibited efflux or influx of ion currents. It was shown that hyperpolarization of endothelial cells occurs in the process of vasodilation and cell relaxation [35], and such processes would probably lead to down regulation of cell stiffness. This was consistent with our cellular characterizations using AFM and fluorescence in the present study (e.g. decreases of Young's modulus and cytoskeleton components). Previous work showed that depolarization could induce decreases in stiffness of endothelial cells [7], whereas, our measurements revealed that hyperpolarization of endothelial cells could be concurrent with down-regulation of cell stiffness. Additionally, DEP-induced membrane electrophysiological dysfunction might be partially ascribed to ROS generation in the context of DEP-cell interaction according to our primary results (Information S6). Though ROS serves as an electron transporter, high chemical reactivity of ROS can also play adverse roles in cells, such as inducing dysfunction of membrane molecules and cell structures from RNA and DNA to proteins and lipids [36]–[38], and even endothelial cell injuries [39].

Our work presented here attempted to elucidate cytotoxic effects of DEPs on VECs via detection of changes in cell behavior and structures. Future work would focus on effects of particular size and chemical compositions on biophysical phenotypes of endothelial cells. By detecting such changes in cell phenotype using nanotechnologies, it could provide access to new knowledge of DEP-induced toxicity for VECs, facilitate better understanding of the biological relevance between changes of biophysical properties and pathological development at the single VEC level in the context of DEPs related urban air-pollution challenging, and eventually help our understanding of DEP-related cardiovascular disorders/diseases.

In the present work, we attempted to evaluate cytotoxicity effects of DEPs on human aortic endothelial cells (HAECs) at single-cell level by analyzing changes of cell topography, membrane nanostructures, cytoskeleton organizations, biomechanics, and alterations of plasma membrane potential (PMP). Fluorescence results evidently revealed that effects of DEP intervention on cell viability appeared in a time- and a dosage-dependent manner. The same alteration tendency of membrane nanostructures and cytoskeleton organizations induced by DEPs was further revealed by AFM and AF/FL. Analyses from flow cytometry indicated that DEP treatment could significantly induce hyperpolarization of endothelial cells that might partially originate from adverse effects of ROS generation. These results implicated that DEP intervention could decrease cell elasticity by down-regulating cytoskeleton components and induce vasodilation. Our findings suggested DEP exposure would trigger important biochemical and biophysical changes that are closely interrelated with pathological developments of cardiovascular disorders or diseases. Our results also provided additional insight into the cytotoxicity of DEPs in HAECs and could facilitate shedding light on mechanisms underlying the development of cardiovascular disorders or diseases associated with DEP-related urban air pollution.

## Supporting Information

Information S1 **SEM of DEP.**
(DOC)

Information S2 **Cell viability.**
(DOC)

Information S3 **AFM images and cell nanomechanics.**
(DOC)

Information S4 **Observations of AF-FL microscopy.**
(DOC)

Information S5 **Bright-field images corresponding to** **Figure 1** **.**
(DOC)

Information S6 **ROS generation measured by flow cytometry.**
(DOC)
