# Supplementary figures and images for: Biophysical Assessment of Single Cell Cytotoxicity: Diesel Exhaust Particle-Treated Human Aortic Endothelial Cells
Source: PLoS One. 2012 May 25;7(5):e36885. doi: 10.1371/journal.pone.0036885 (PMC3360744; doi:10.1371/journal.pone.0036885)

| 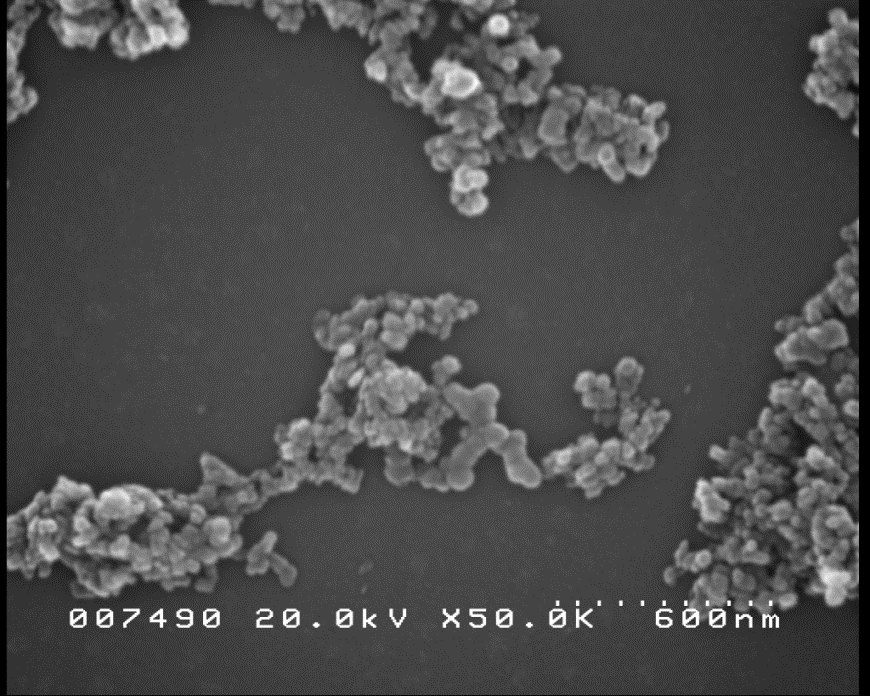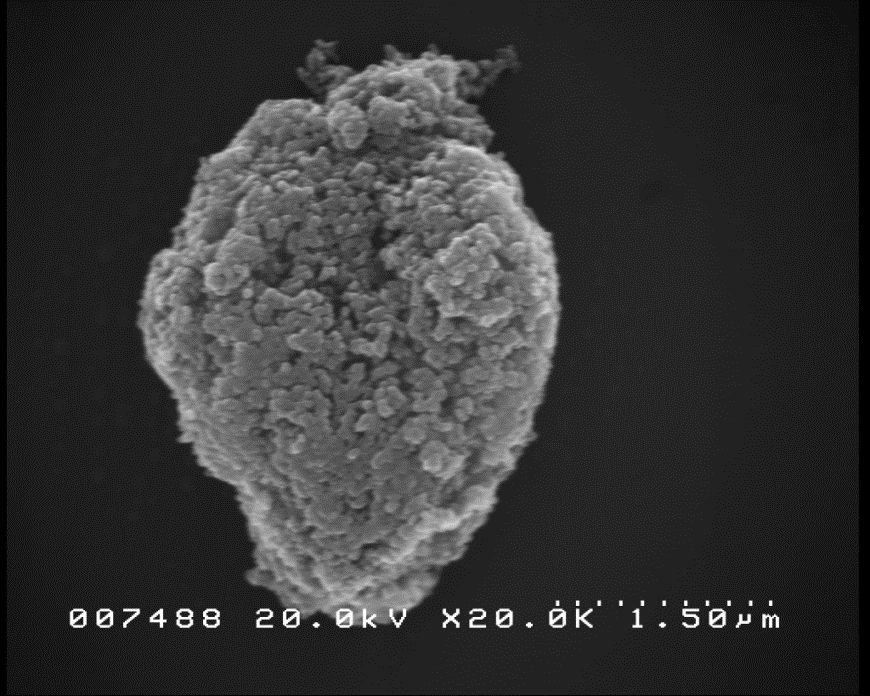 |
| --- |

**Figure S1-1**. Representative images of DEP acquired by Hitachi S-4000 scanning electron microscopy.

Supplement: Information S1 — SEM of DEP. (DOC) [file pone.0036885.s001.doc]
