# Supplementary material for: Biophysical Assessment of Single Cell Cytotoxicity: Diesel Exhaust Particle-Treated Human Aortic Endothelial Cells
Source: PLoS One. 2012 May 25;7(5):e36885. doi: 10.1371/journal.pone.0036885 (PMC3360744; doi:10.1371/journal.pone.0036885)
Supplement: Information S2 — Cell viability. (DOC) [file pone.0036885.s002.doc]

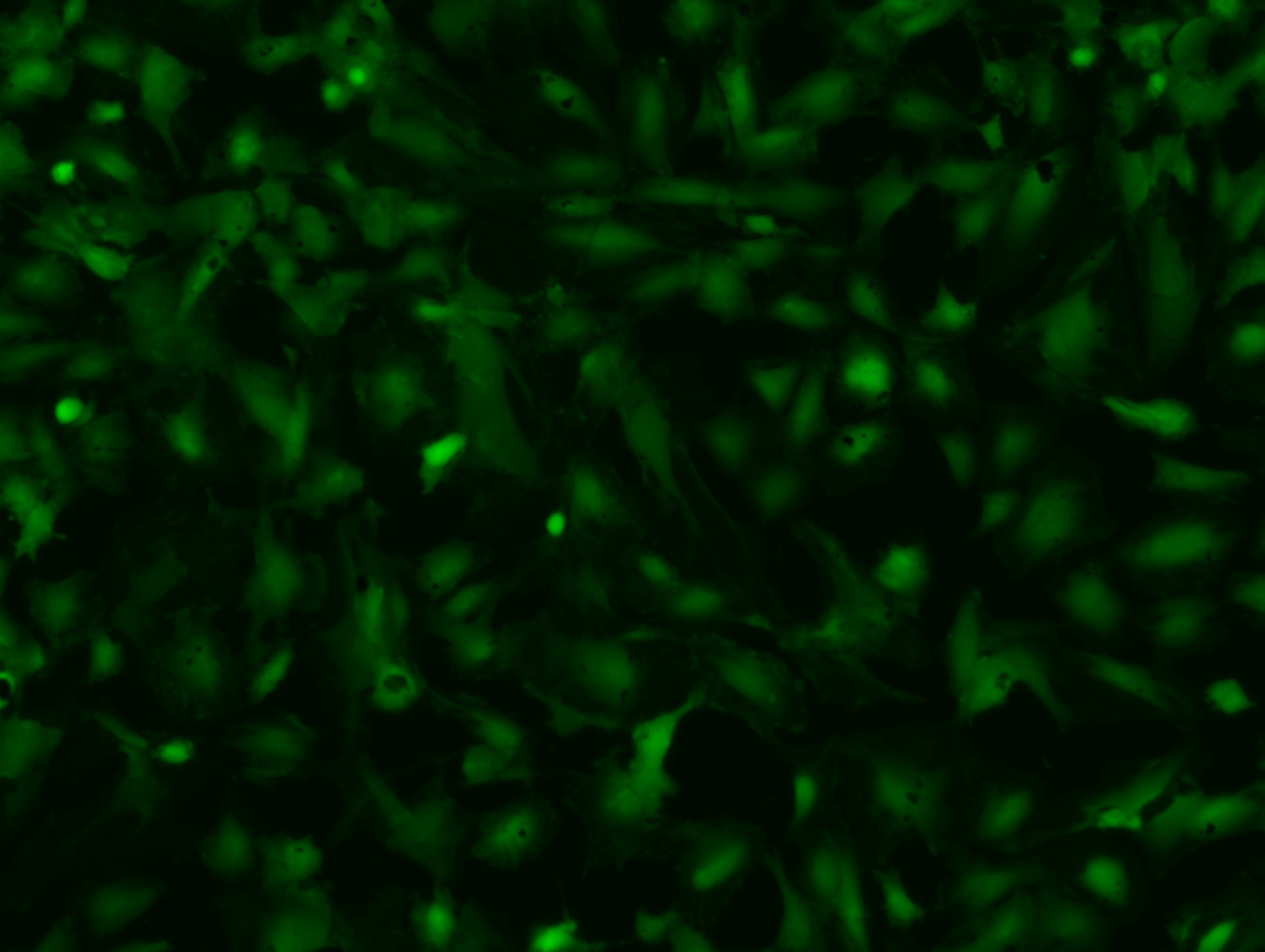


**Figure S2-1.** Representative fluorescence images of cell viability of unexposed live HAECs. Cells are stained with Invitrogen LIVE/DEAD Viability/Cytotoxicity Assay Kit. Green fluorescence means live cells. Image was obtained with 10× lens.

| 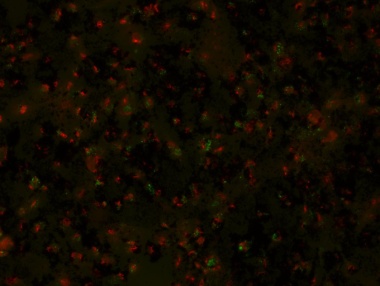 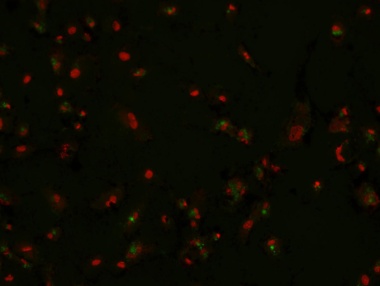 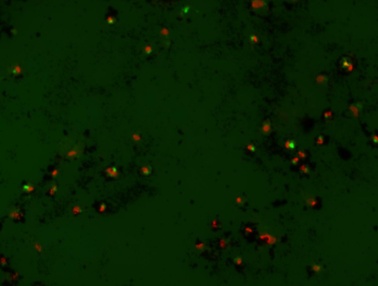 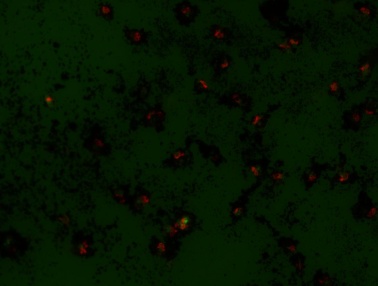  **4 hours 8 hours 24 hours 48 hours** |
| --- |

**Figure S2-2.** Representative fluorescence images of cell viability of **1000 µg/ml DEP** exposed HAECs. Cells are stained with Invitrogen LIVE/DEAD Viability/Cytotoxicity Assay Kit. Green fluorescence means live cells, whereas red fluorescence indicates dead/damaged cells. Image was obtained with 10× lens. In this group, only a few cells could be seen after incubation for 24 and 48 hours, implying that long-term exposure of a high concentration of DEPs could induce cell death and cell shape destruction.
