# Supplementary material for: Biophysical Assessment of Single Cell Cytotoxicity: Diesel Exhaust Particle-Treated Human Aortic Endothelial Cells
Source: PLoS One. 2012 May 25;7(5):e36885. doi: 10.1371/journal.pone.0036885 (PMC3360744; doi:10.1371/journal.pone.0036885)
Supplement: Information S3 — AFM images and cell nanomechanics. (DOC) [file pone.0036885.s003.doc]

| **4 hours**  **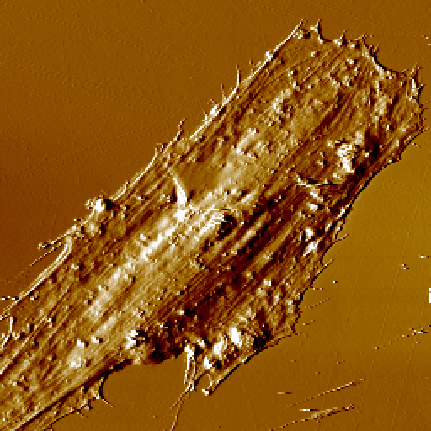**  **86 µm**  **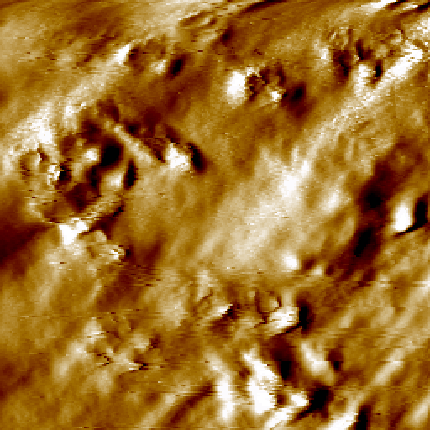**  **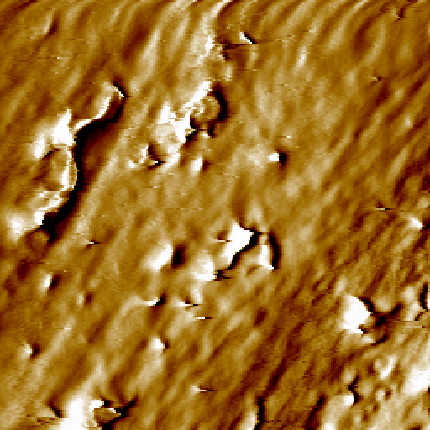** | **8 hours**  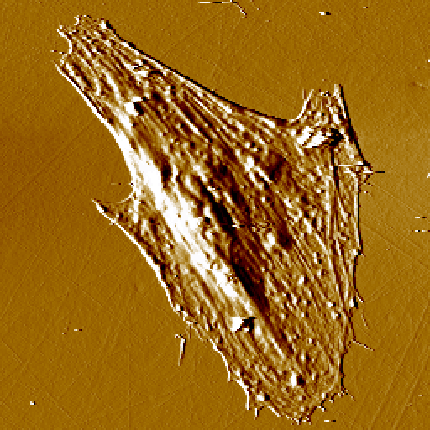  **80 µm**  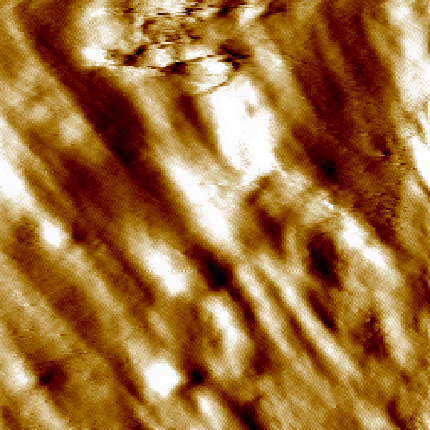  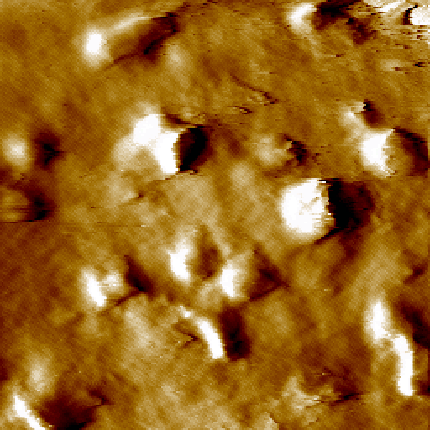 | **24 hours**  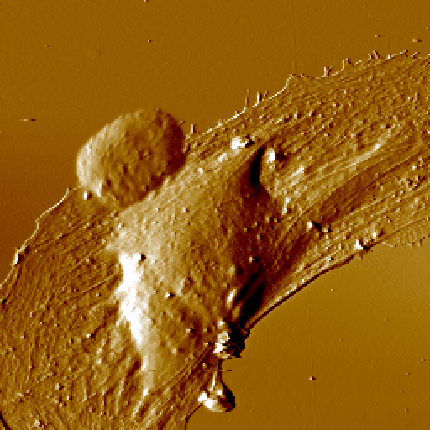  **80 µm**  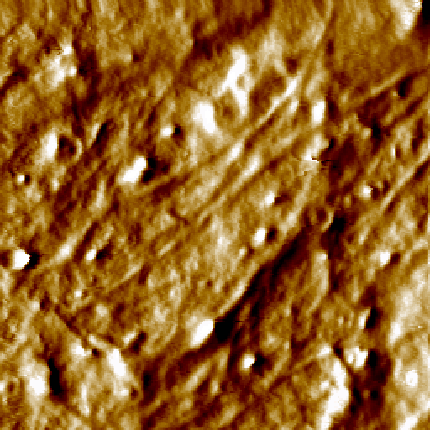  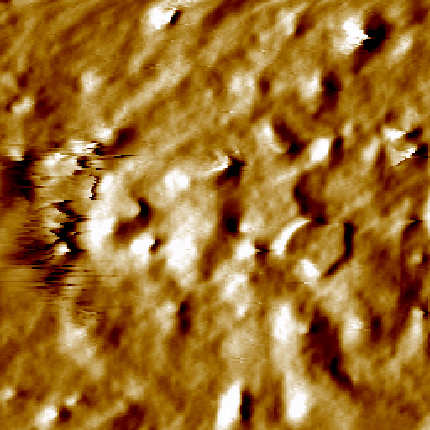 | **48 hours**  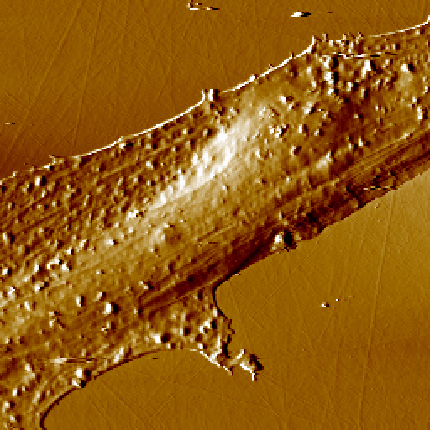  **60 µm**  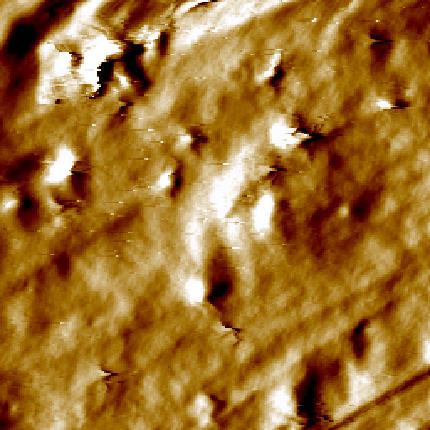  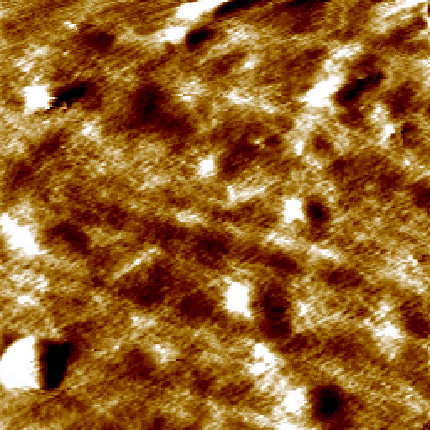 |
| --- | --- | --- | --- |

**Figure S3-1.** Representative AFM deflection mode (this mode image is acquired based on the “error signal” of the position-sensitive photodiode detector resulting from the changes of laser spot position corresponding to probe cantilever bending; this mode usually can show structural details much more well than ‘height’ mode, especially for cell samples with large height) images of **50 µg/ml of DEPs** treated HAECs observed in PBS. Representative AFM deflection mode images of 50 µg/ml of DEPs treated HAECs (cells were fixed prior to observe in PBS). Column 1 shows images of cells treated with DEPs for 4 hours; column 2, 8 hours; column 3, 24 hours; column 4, 48 hours. Row 1 shows deflection mode images of single cells; rows 2 and 3 show images of membrane surface ultrastructures. DEP particles on cells are readily seen. Scanning size of row 1 is marked on respective image; and that of row 2 and row 3 (ultrastructures): 10 µm × 10 µm.

| **4 hours**  **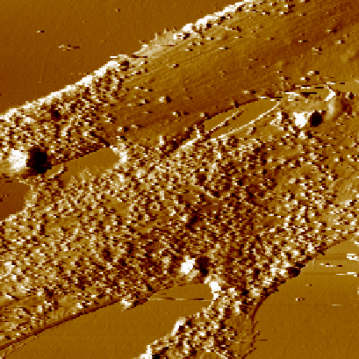**  **70 µm**  **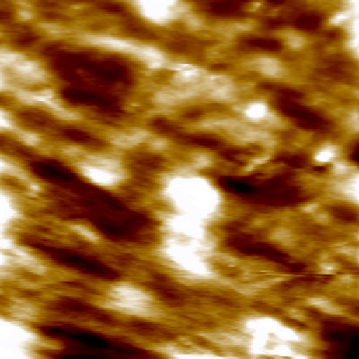**  **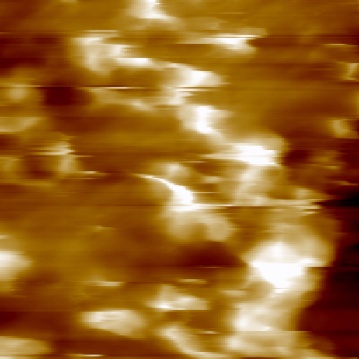** | **8 hours**  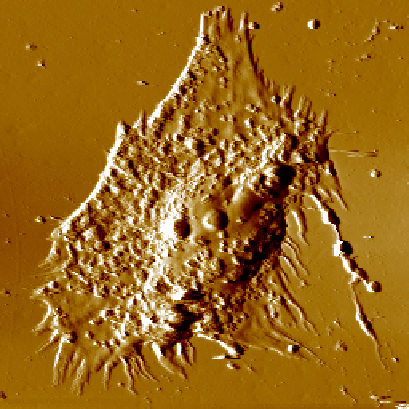  **80 µm**  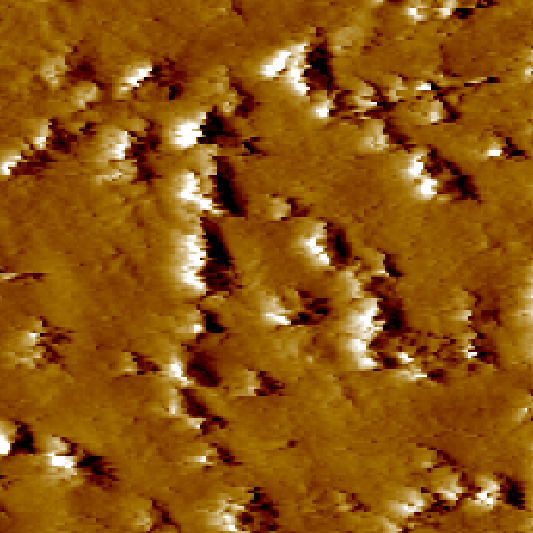  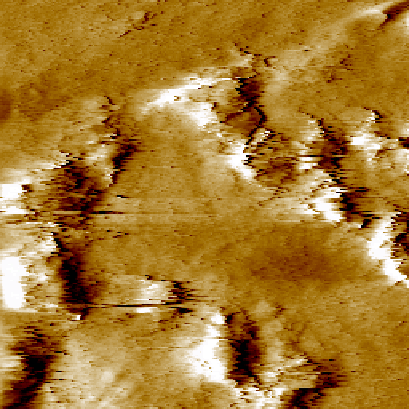 | **24 hours**  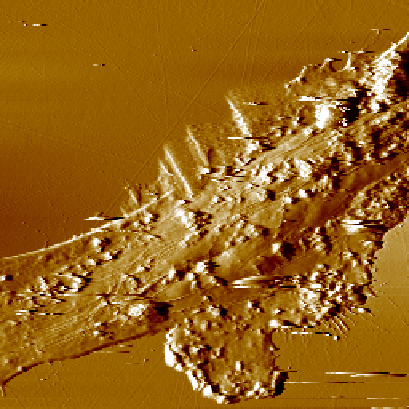  **60 µm**  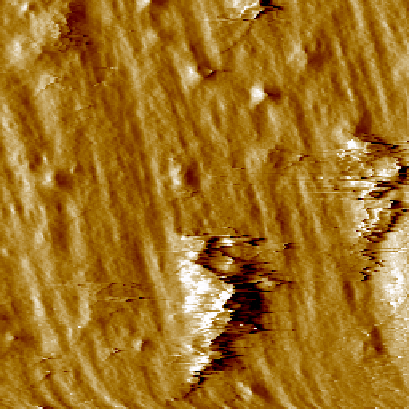  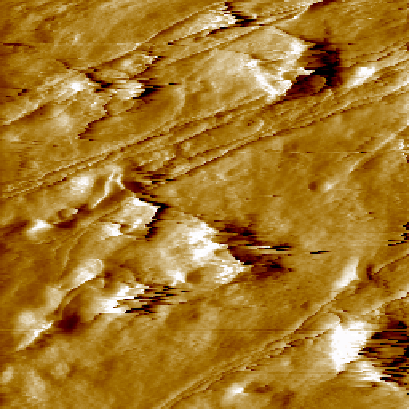 | **48 hours**  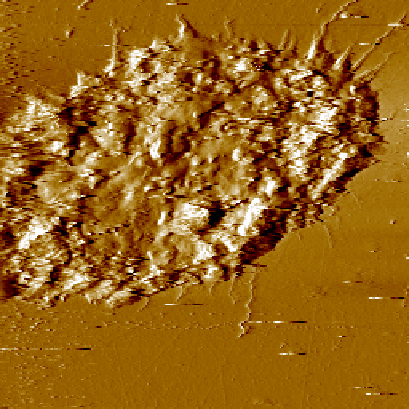  **60 µm**  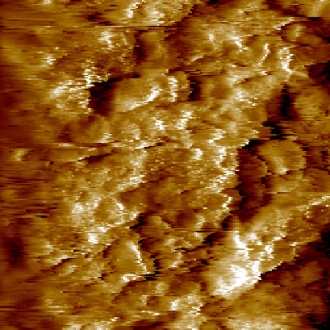  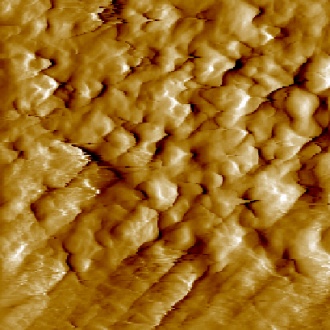 |
| --- | --- | --- | --- |

**Figure S3-2.** Representative AFM deflection mode images of **100 µg/ml of DEPs** treated HAECs acquired in PBS. Image panel arrangement corresponds to **Fig S-1**. Scanning size of row 2 and row 3 (ultrastructures): 10 µm × 10 µm.

| **4 hours**  **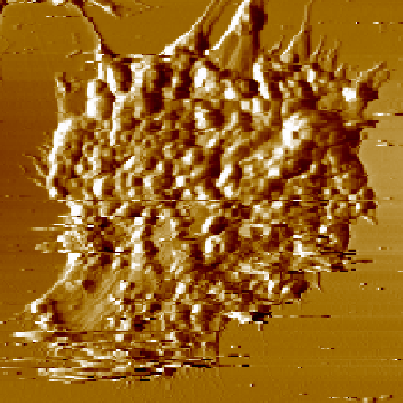**  **60 µm**  **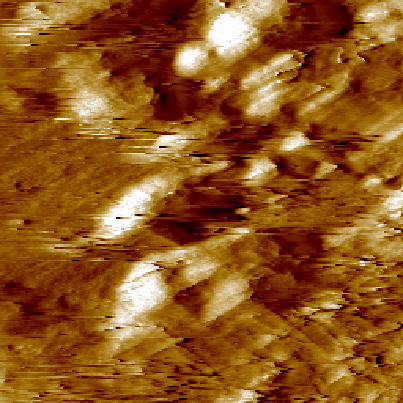**  **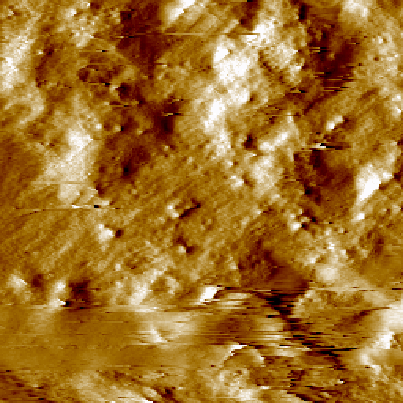** | **8 hours**  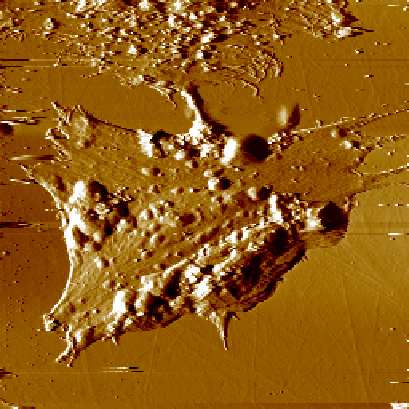  **60 µm**  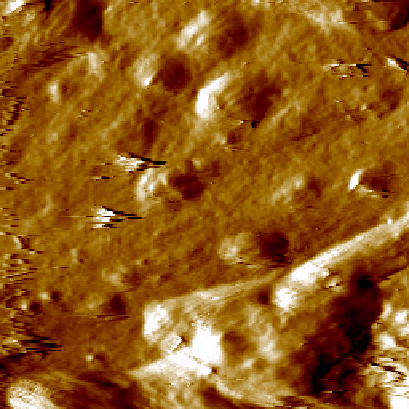  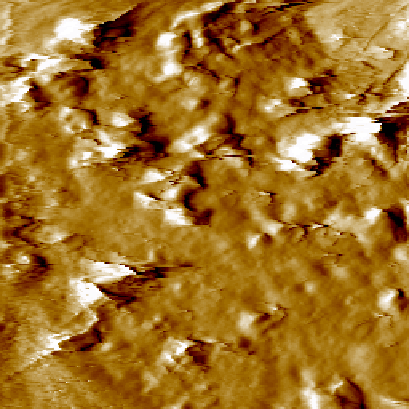 | **24 hours**  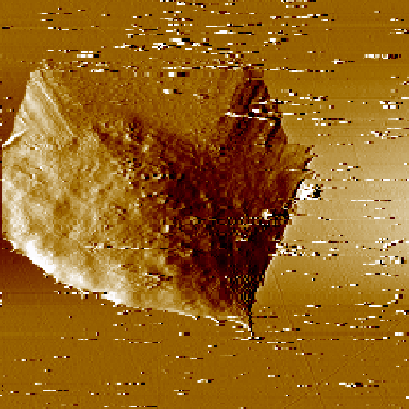  **70 µm**  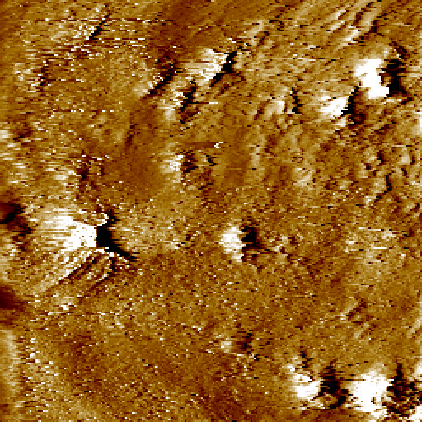  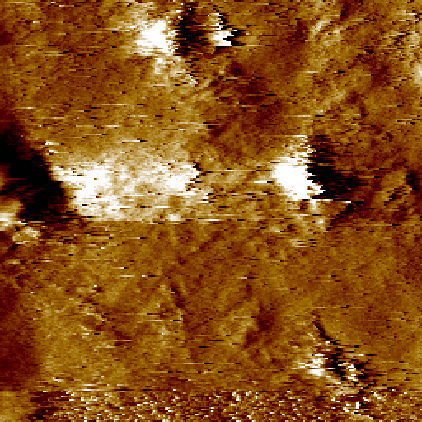 | **48 hours**  NO IMAGE ACQUIRED |
| --- | --- | --- | --- |

**Figure S3-3.** Representative AFM deflection mode images of **1000 µg/ml of DEPs** treated HAECs acquired in PBS. Image panel arrangement corresponds to **Fig S-1**. Scanning size of row 2 and row 3 (ultrastructures): 10 µm × 10 µm. It should be noted here that, after 48 hours of DEPs treatment, it become very difficult to locate cells using video camera or bright field microscopy, because almost all cells were destroyed by this high concentration of DEPs, thereby AFM images of the group of 48 hours were not presented in this figure panel. Images of this group clearly indicated that cell membrane surface is covered by large amount of DEPs. Notably, when cells were treated with 1000 μg/ml DEPs, neither AFM images nor fluorescence images (**Fig. S7**) show visible cytoskeleton, especially after 24 and 48 hours of treatment.

| **(I) 10 µg/ml DEP**  **8**  **24**  **48**  **70**  **70**  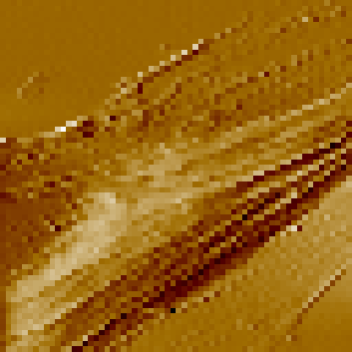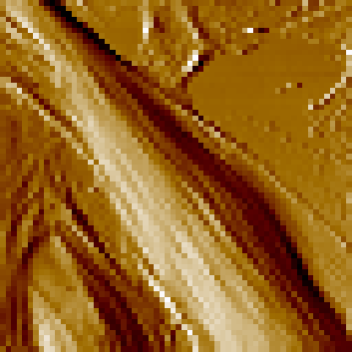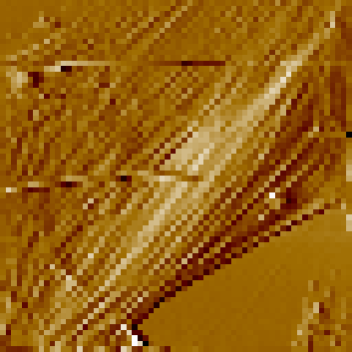  **60**  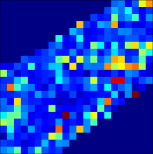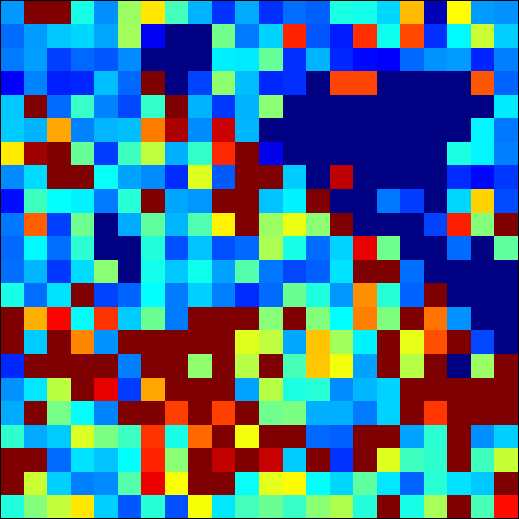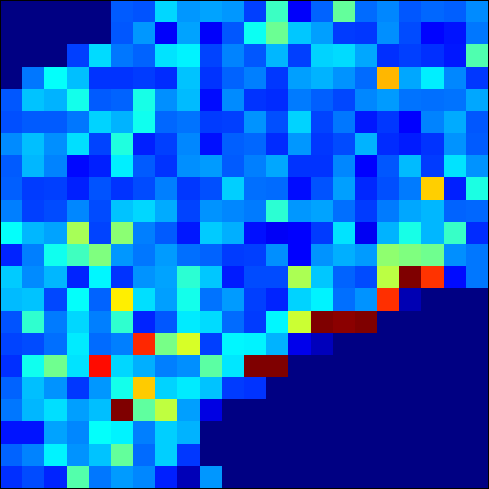  ***E* maps *F_ad_* maps**  ***E* maps *F_ad_* maps**  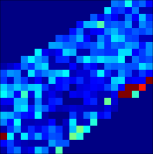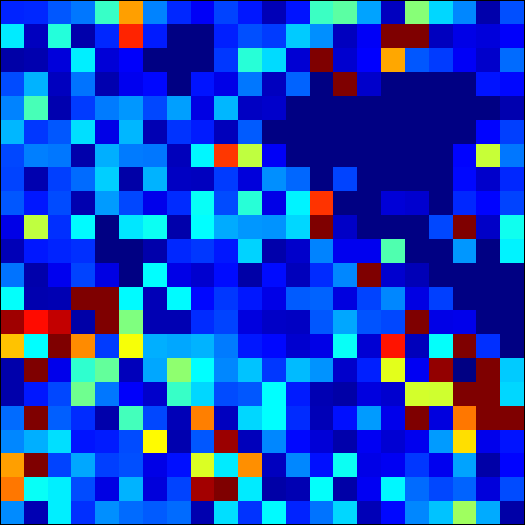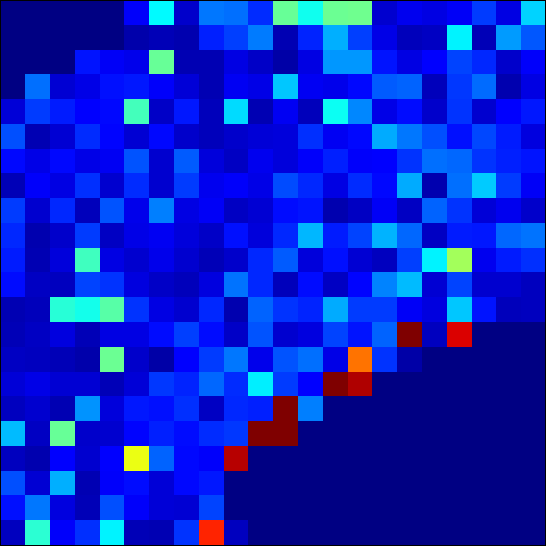 | **(II) 50 µg/ml DEP**  **hours**  **8**  **24**  **48**  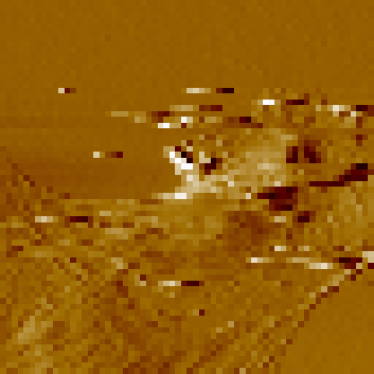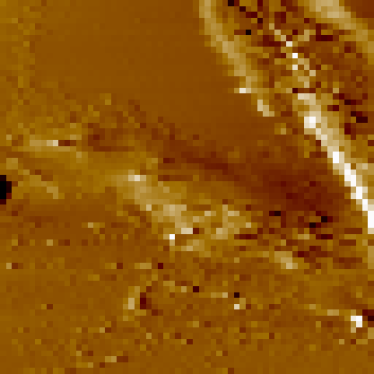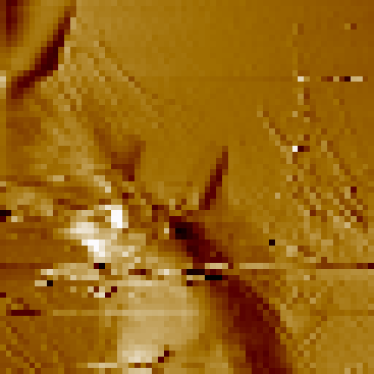  **nN**  **kPa**  **µm**  **60**  **60**  **60**  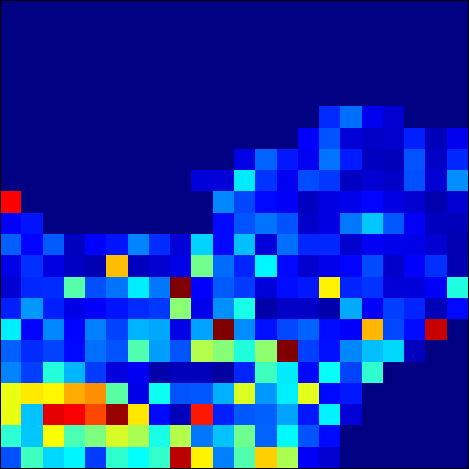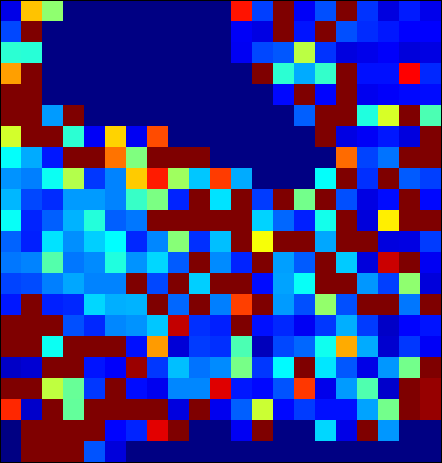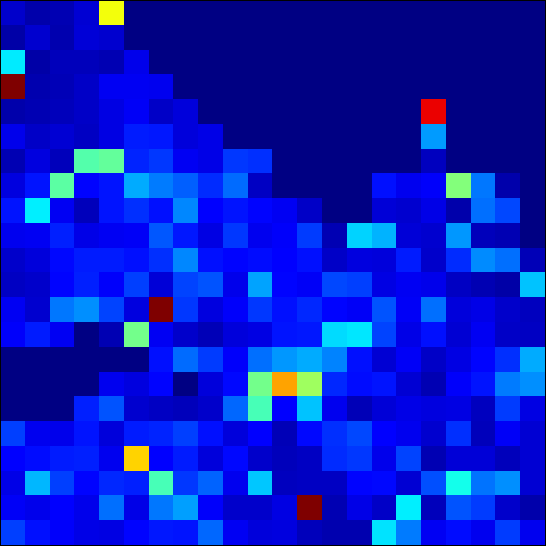 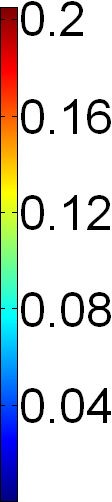  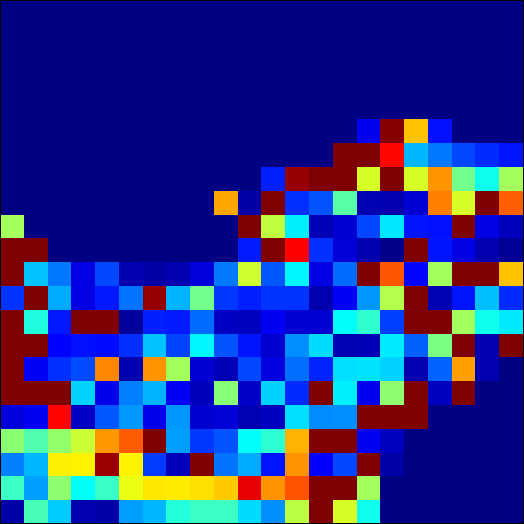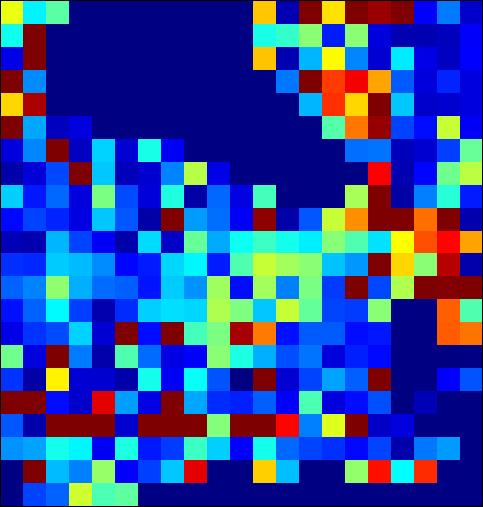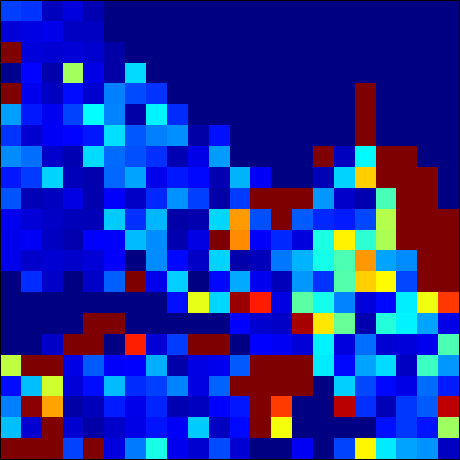 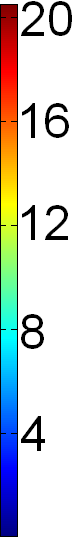 |
| --- | --- |
| **(III) 100 µg/ml DEP**  **8**  **24**  **48**  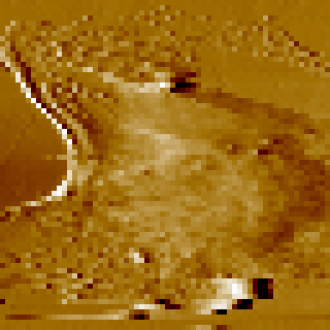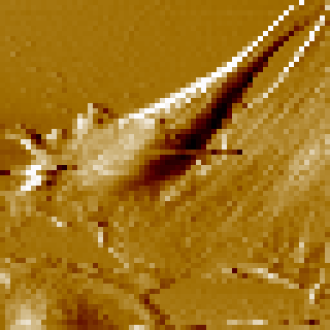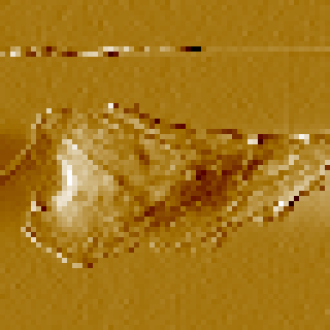  **70**  **60**  **60**  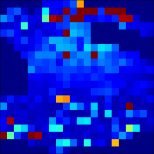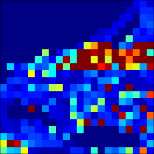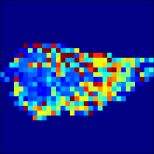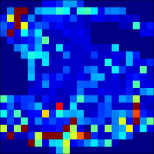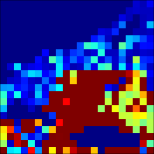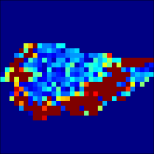 | **(IV) 1000 µg/ml DEP**  **4**  **8**  **24**  **hours**  **48**  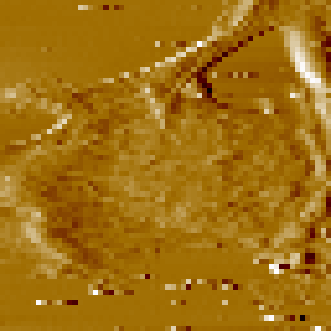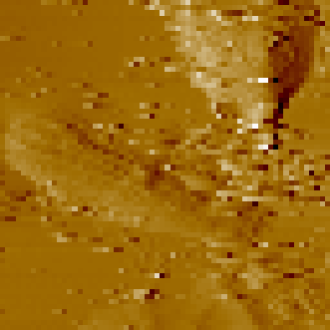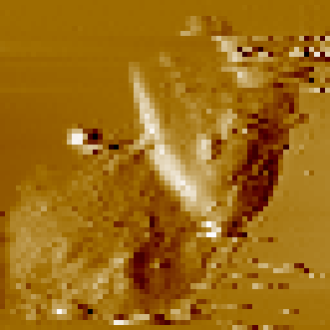  **nN**  **kPa**  No image acquired  **80**  **60**  **µm**  **70**  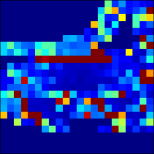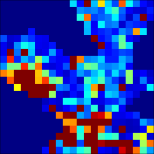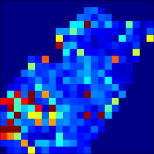  **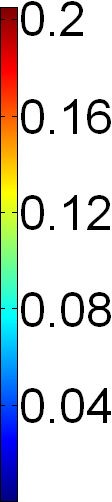**  No data acquired  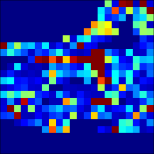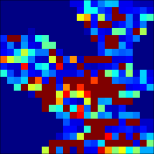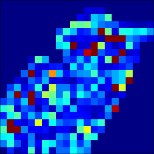 **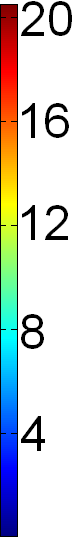** |

**Figure S3-4**. Measurements of cell mechanics (adhesion force, F_ad_; Young’s modulus, E) of live HAECs treated with DEPs for different exposure times and various concentrations. Panels I-IV, mechanical measurements of HAECs treated by 10 μg/ml, 50 µg/ml, 100 µg/ml and 1000 µg/ml of DEPs, respectively. The color bars showing at the right of maps display the value scale of adhesion force (*F*_ad_, nN) and Young’s modulus (*E,* kPa). The scanning size (µm) of AFM images and DEP–cell interaction time are marked on each image. For group IV, after cells were treated for 48 hours, it’s hardly to obtain AFM image of a cell, therefore, no image data shown here.
