# Supplementary material for: Biophysical Assessment of Single Cell Cytotoxicity: Diesel Exhaust Particle-Treated Human Aortic Endothelial Cells
Source: PLoS One. 2012 May 25;7(5):e36885. doi: 10.1371/journal.pone.0036885 (PMC3360744; doi:10.1371/journal.pone.0036885)
Supplement: Information S4 — Observations of AF-FL microscopy. (DOC) [file pone.0036885.s004.doc]

| 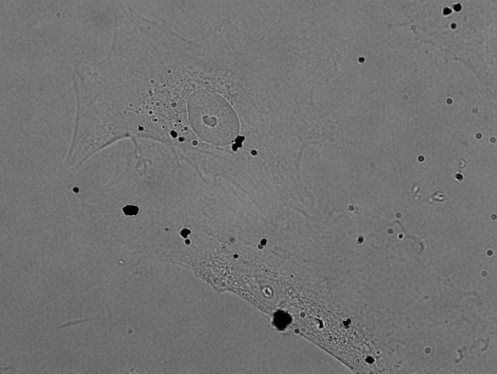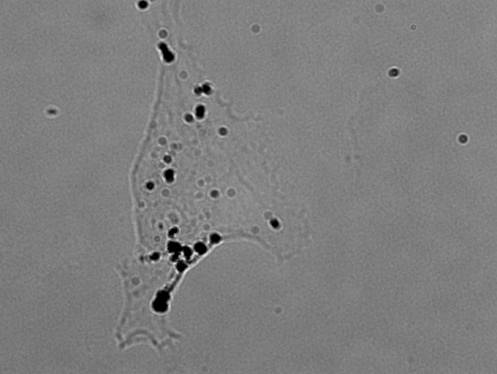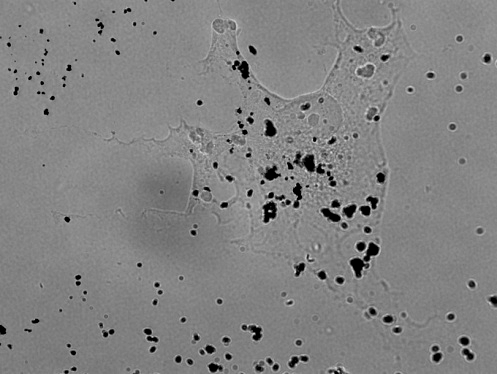  **4 hrs 8 hrs 24 hrs 48 hrs** |
| --- |
| 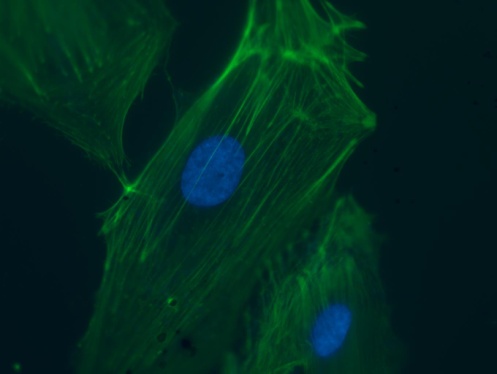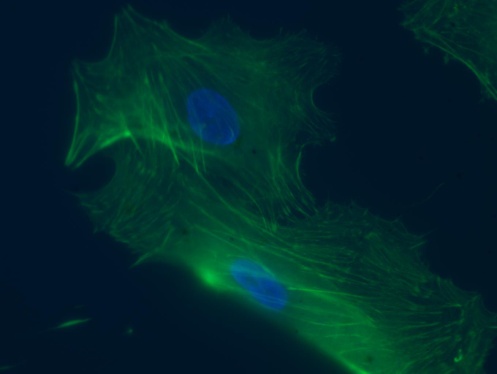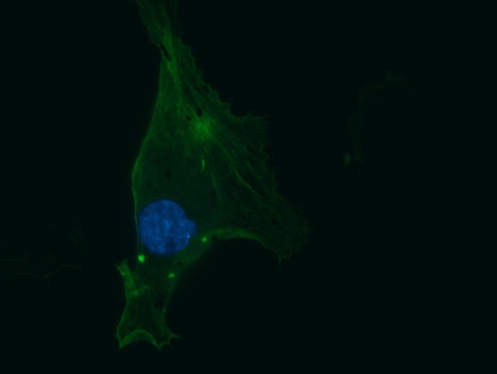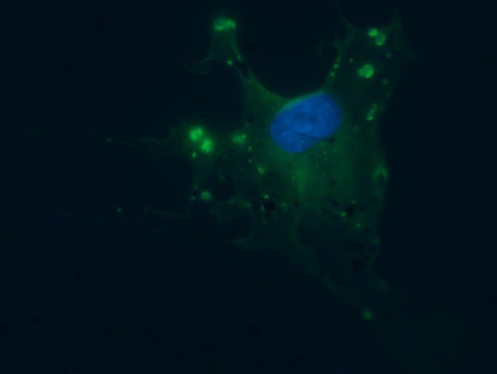 |
| 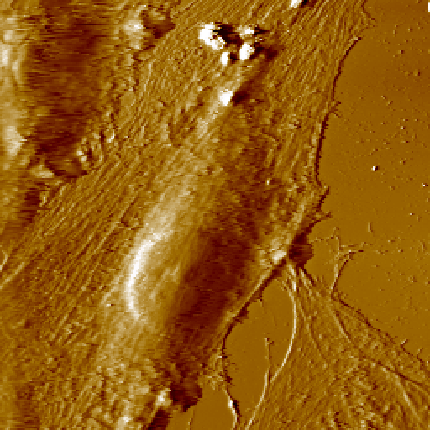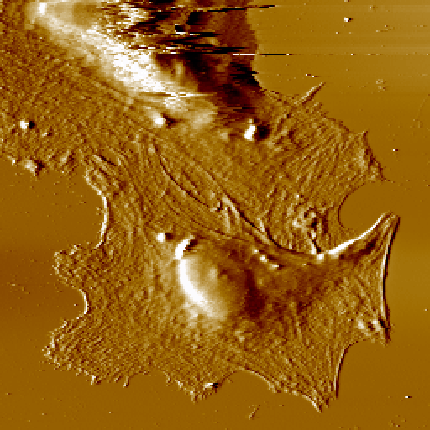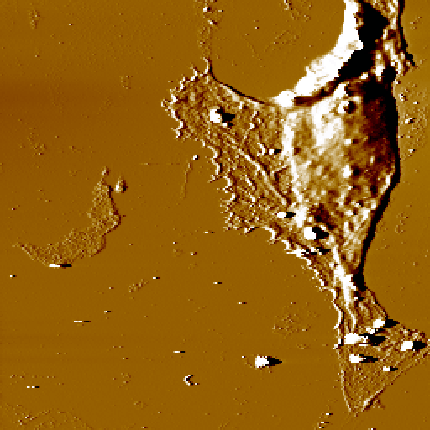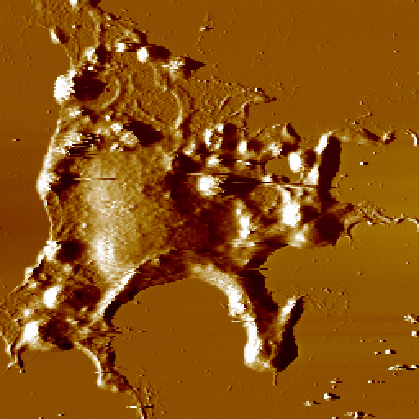  **80 µm**  **90 µm**  **90 µm**  **90 µm** |

**Figure S4-1.** Representative observations of DEP (**50 µg/ml**) -treated HAECs (fixed cells) in PBS using the coupled AF/FL microscope. Image panel arrangement corresponds to **Fig. S-5**. Optical images were obtained by using a 60× oil objective. For this group, AF/FL observations indicated that cytoskeletons became increasingly obscure with increases of exposure time; and after 24 hours of treatment, cell shape and cell architectures especially lamellipodia structures became obviously contracted.

| 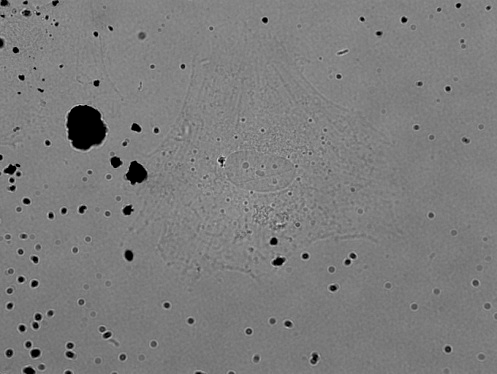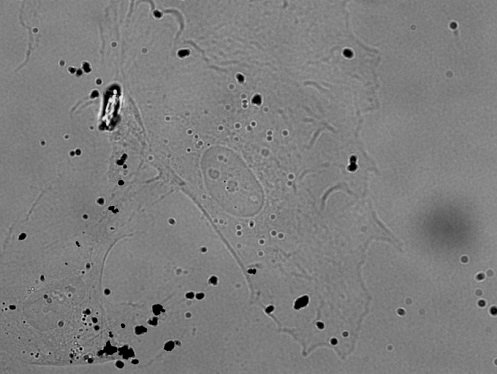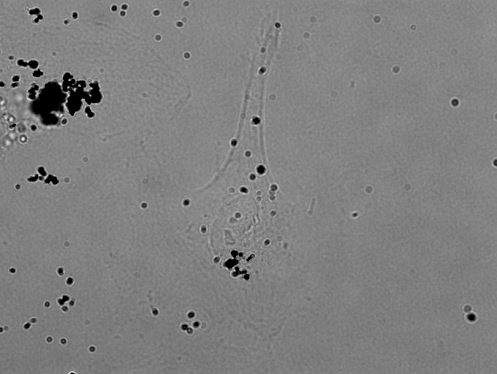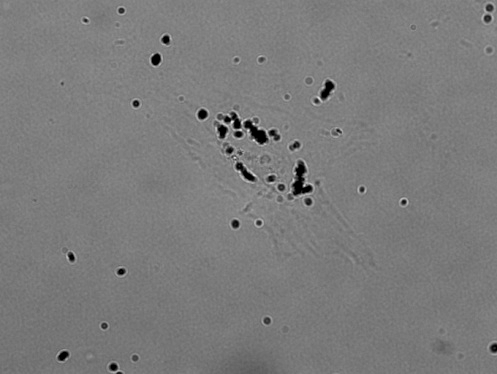  **4 hrs 8 hrs 24 hrs 48 hrs** |
| --- |
| 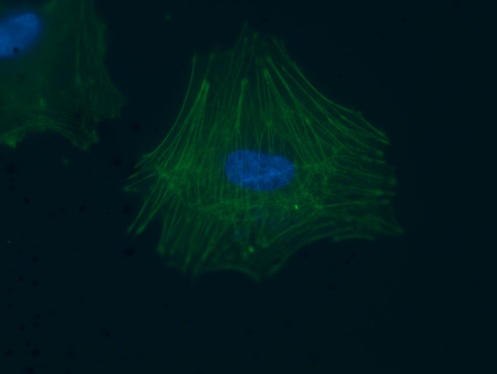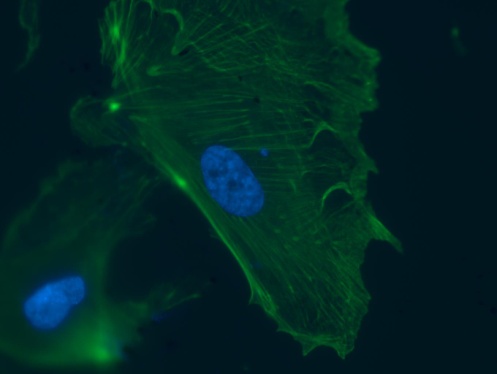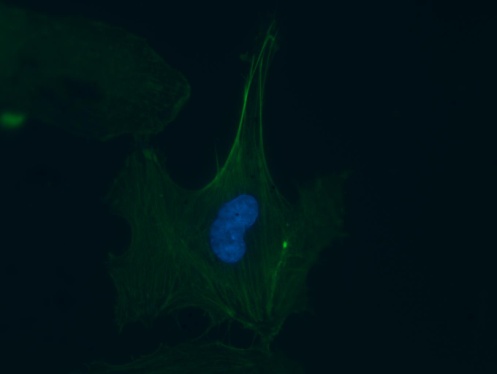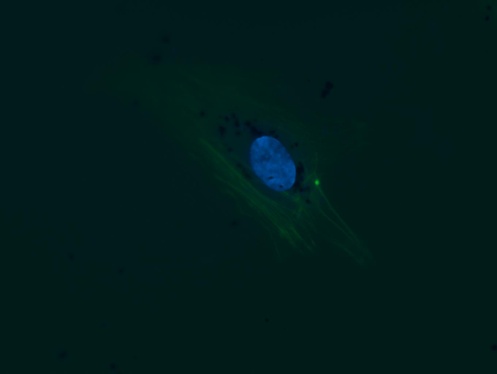 |
| 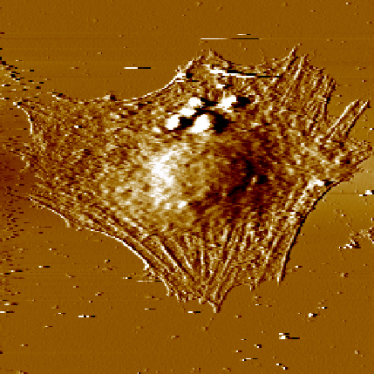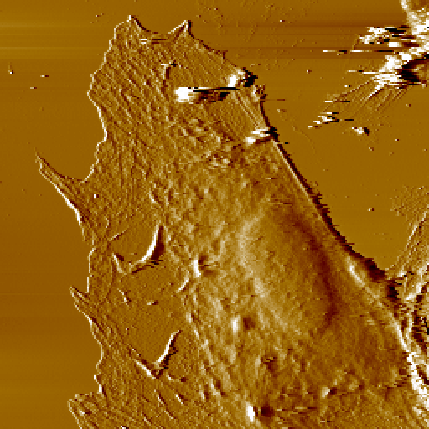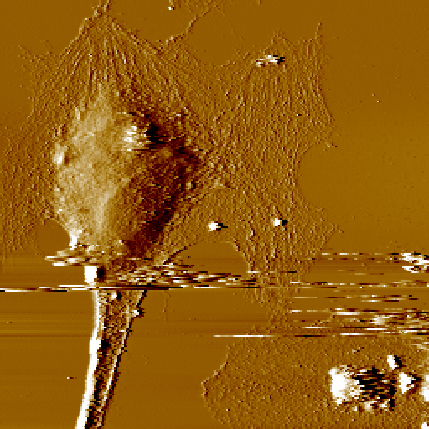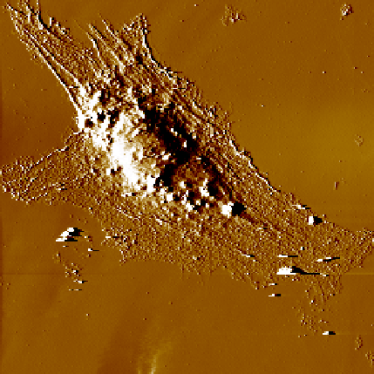  **80 µm**  **80 µm**  **80 µm**  **80 µm** |

**Figure S4-2.** Representative visualizations of DEP (**100 µg/ml**) -treated HAECs (fixed cells) in PBS using the coupled AF/FL microscope. Image panel arrangement also corresponds to **Fig. S-5**. Optical images were obtained using a 60× oil objective. After cells were treated with 100 µg/ml DEPs, images exhibited similar alterations to **Figure S-6**, but cytoskeletal structures were impaired more severely, suggesting that a higher dosage of DEPs leads to gradual down-regulation of cytoskeleton components. Especially after 48 hours of treatment, it appeared that cell shape was hardly recognized from fluorescence image, indicating cellular cytoskeleton was largely destructed.

| 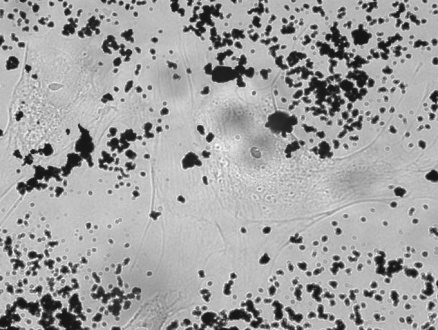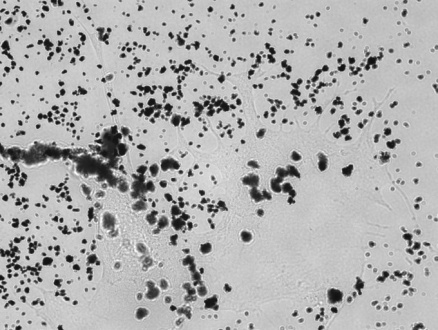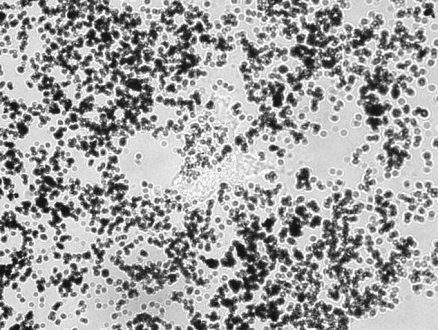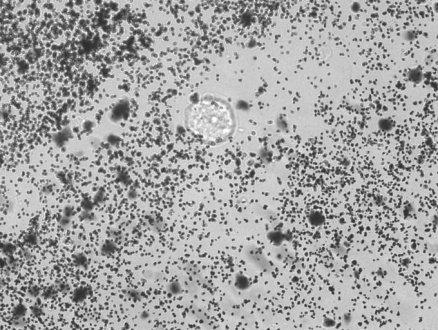  **4 hrs 8 hrs 24 hrs 48 hrs** |
| --- |
| 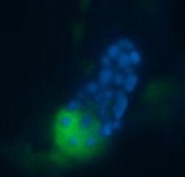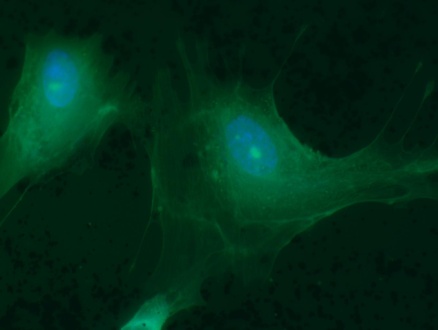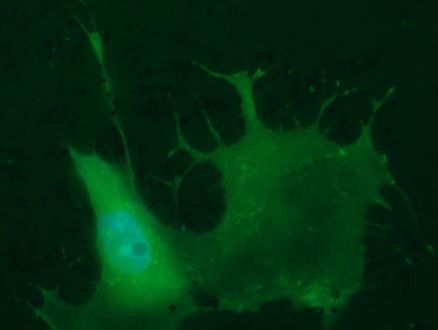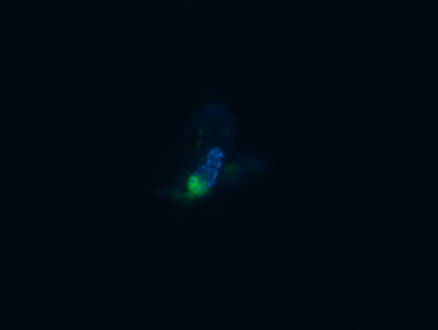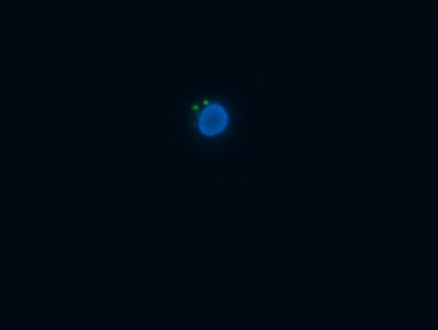 |

**Figure S4-3.** Representative images of DEP (**1000 μg/ml**) -treated HAECs (fixed cells) in PBS acquired by the fluorescence microscope only. Row 1 shows bright-field images, and row 2 contains fluorescent images. Optical images were obtained using a 60× oil objective. The inset in column 3 (fluorescence) is an enlarged view of broken cell nucleus (blue, DABI). For this group, after this high concentration of DEP treatment, only very few cells remained on the substrate, and it became very hard to visualize or locate using optical microscope and could only be seen by fluorescence imaging due to the large amount of DEP covering these cells, therefore, no AFM image was acquired here. Fluorescence images indicated that cytoskeletal structures (green, phalloidin) were almost destroyed at the first 4 hours of exposure. After 24 hours or 48 hours of DEP treatment, no green fluorescence was seen, indicating the cellular cytoskeleton was completely destroyed. It is worth noting that high concentration of DEP intervention could destroy not only cytoskeletal structures but also cell nucleus (columns 3 and 4). With increases of DEP concentration and exposure time, the gradual shrinkage in size of cell nucleus (**Figs. 6, 7; Figs. S5-S6**) and ultimate destruction (**Fig. S7**) of the cell nucleus was evident.
