# Supplementary material for: Biophysical Assessment of Single Cell Cytotoxicity: Diesel Exhaust Particle-Treated Human Aortic Endothelial Cells
Source: PLoS One. 2012 May 25;7(5):e36885. doi: 10.1371/journal.pone.0036885 (PMC3360744; doi:10.1371/journal.pone.0036885)
Supplement: Information S5 — Bright-field images corresponding to Figure 1 . (DOC) [file pone.0036885.s005.doc]

**DEP : 100 µg/ml 50 µg/ml 10 µg/ml**

| 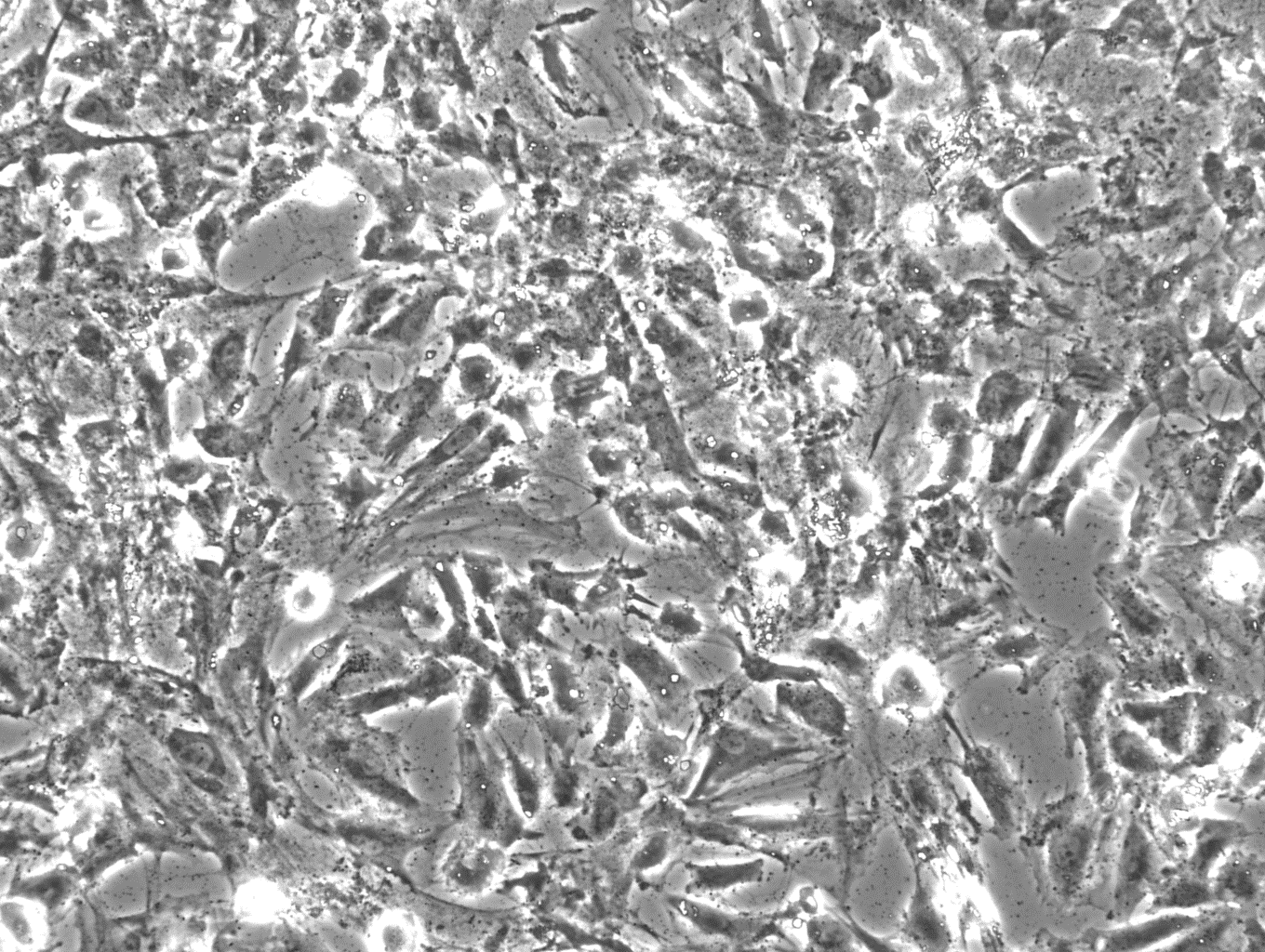 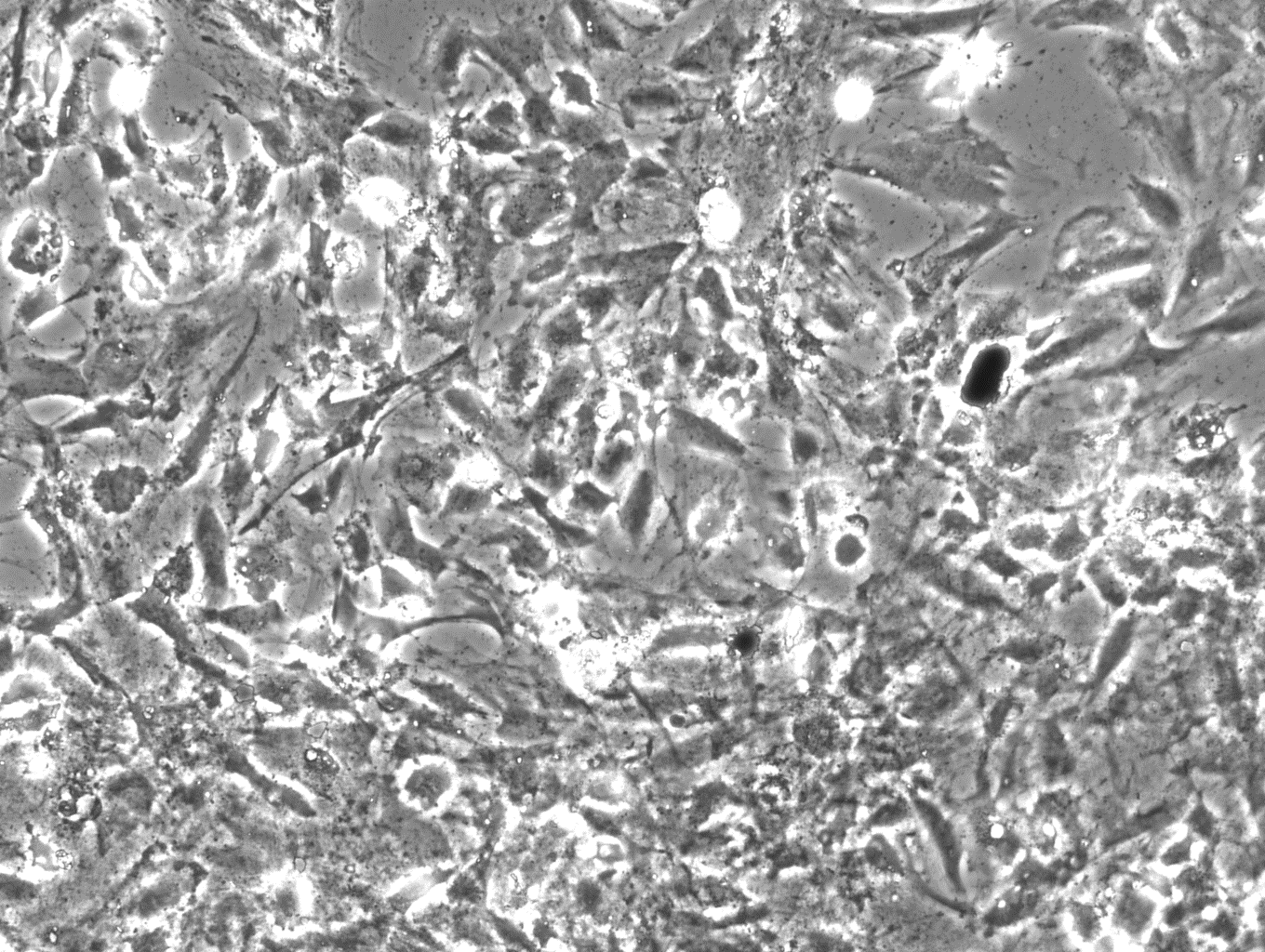 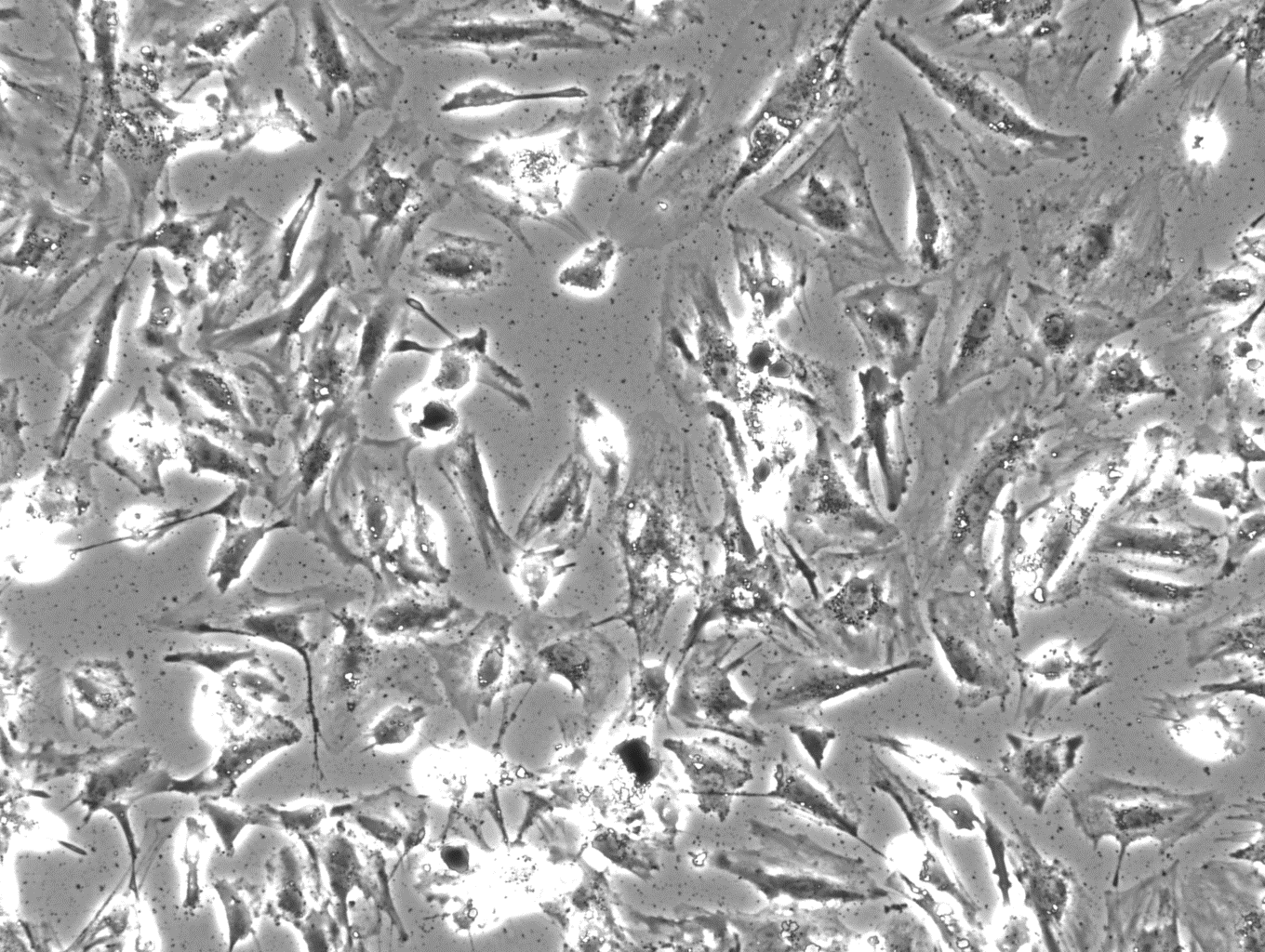 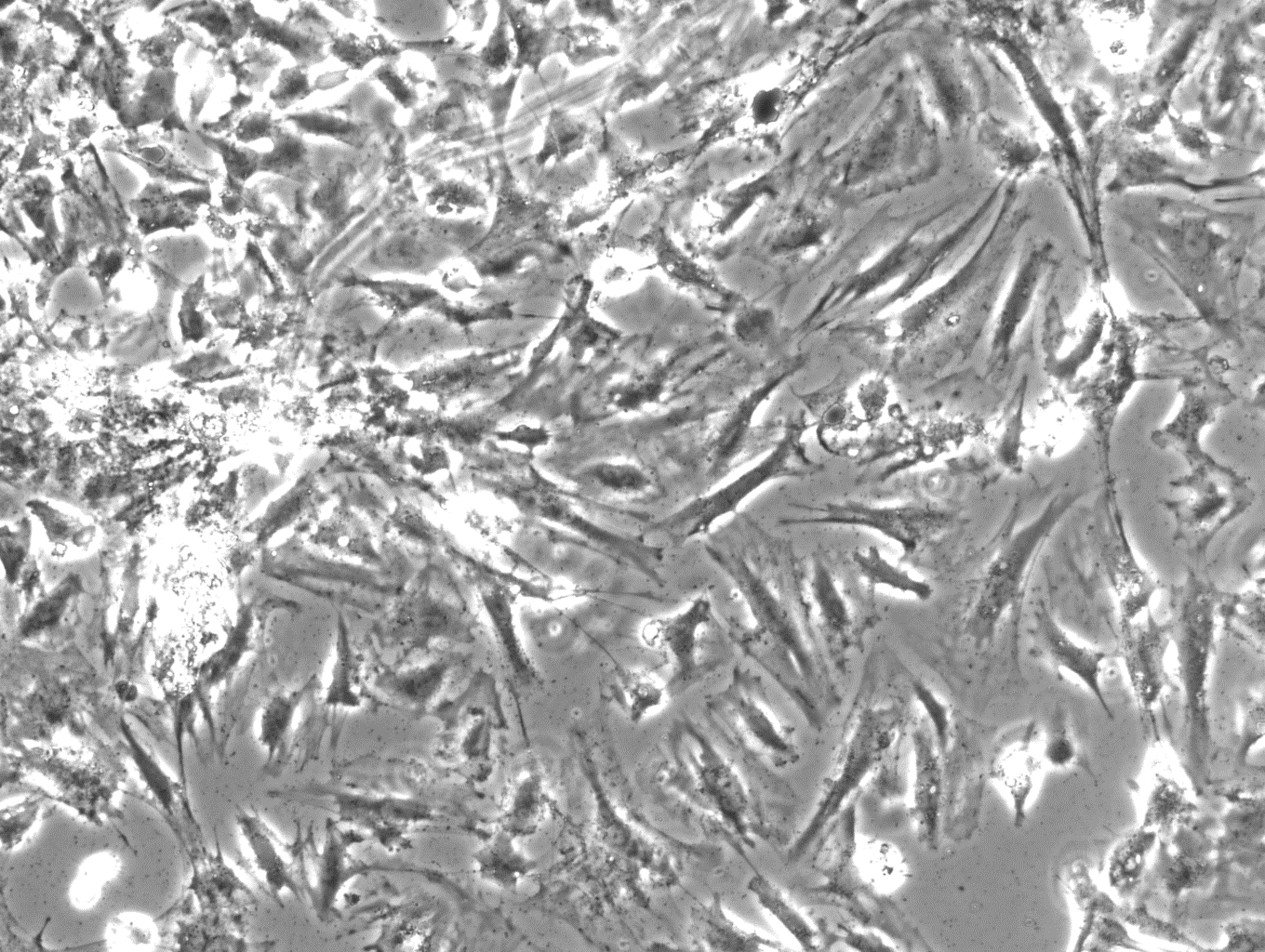  **4 hours 8 hours 24 hours 48 hours** |
| --- |
| 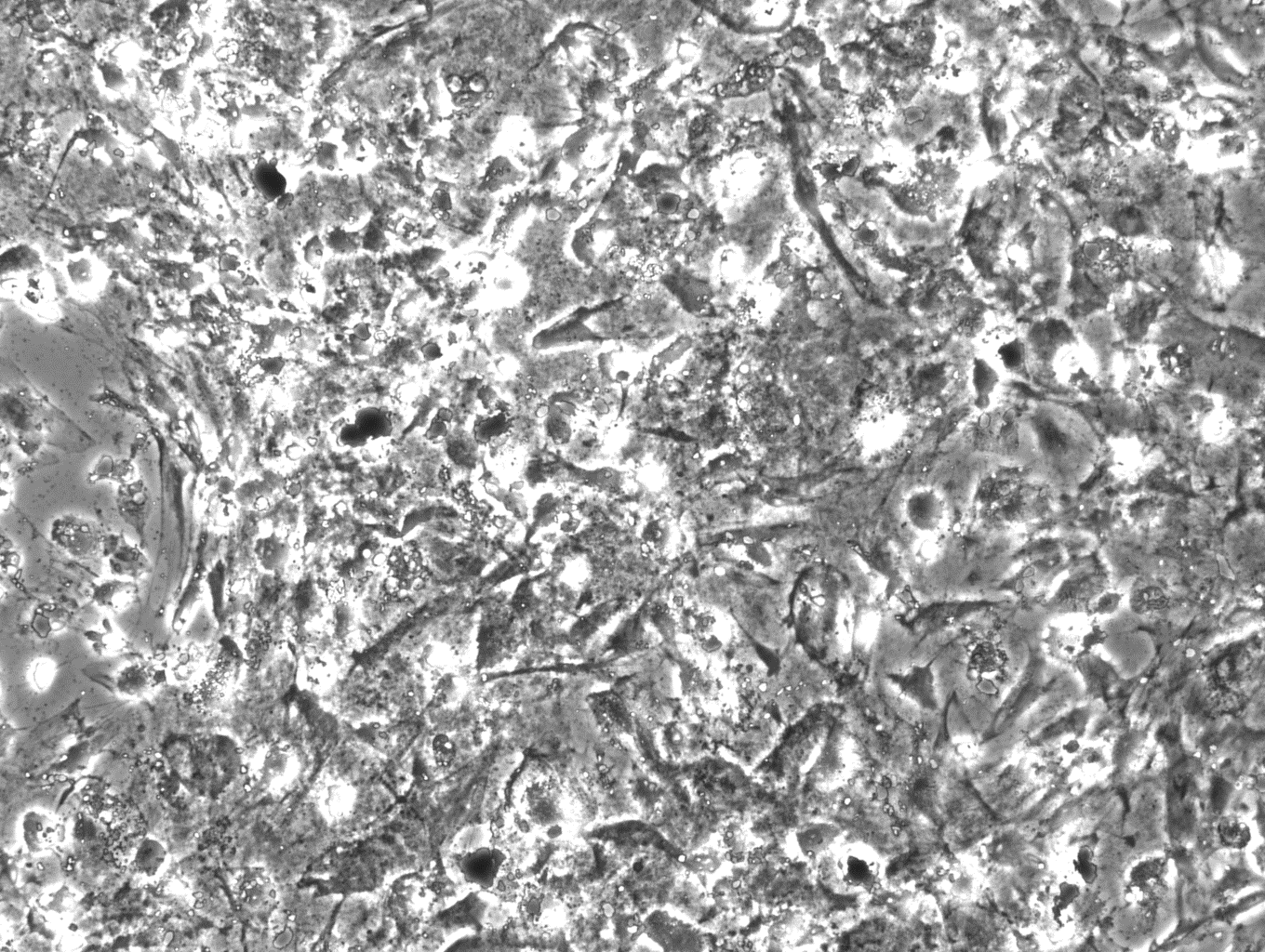 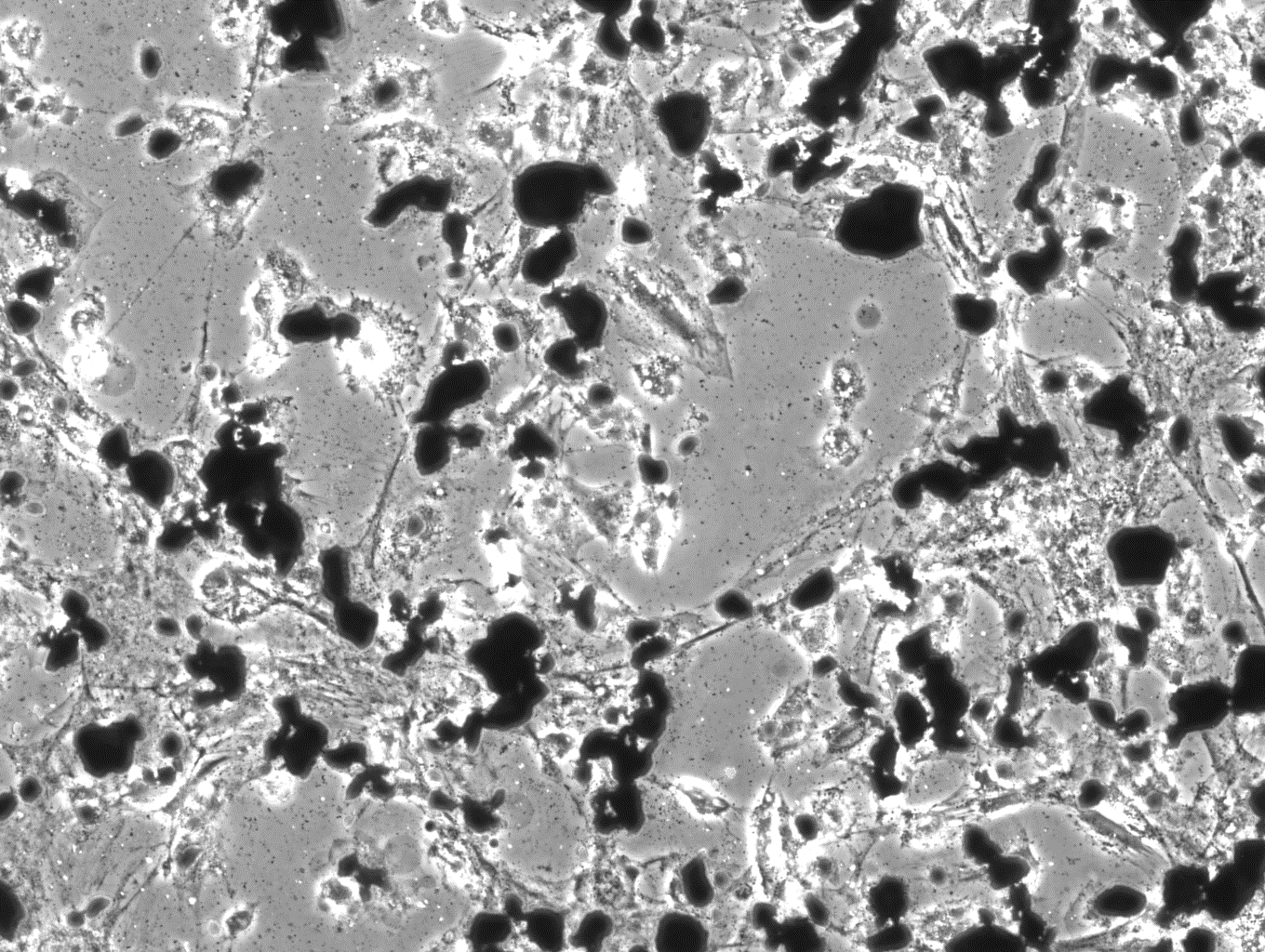 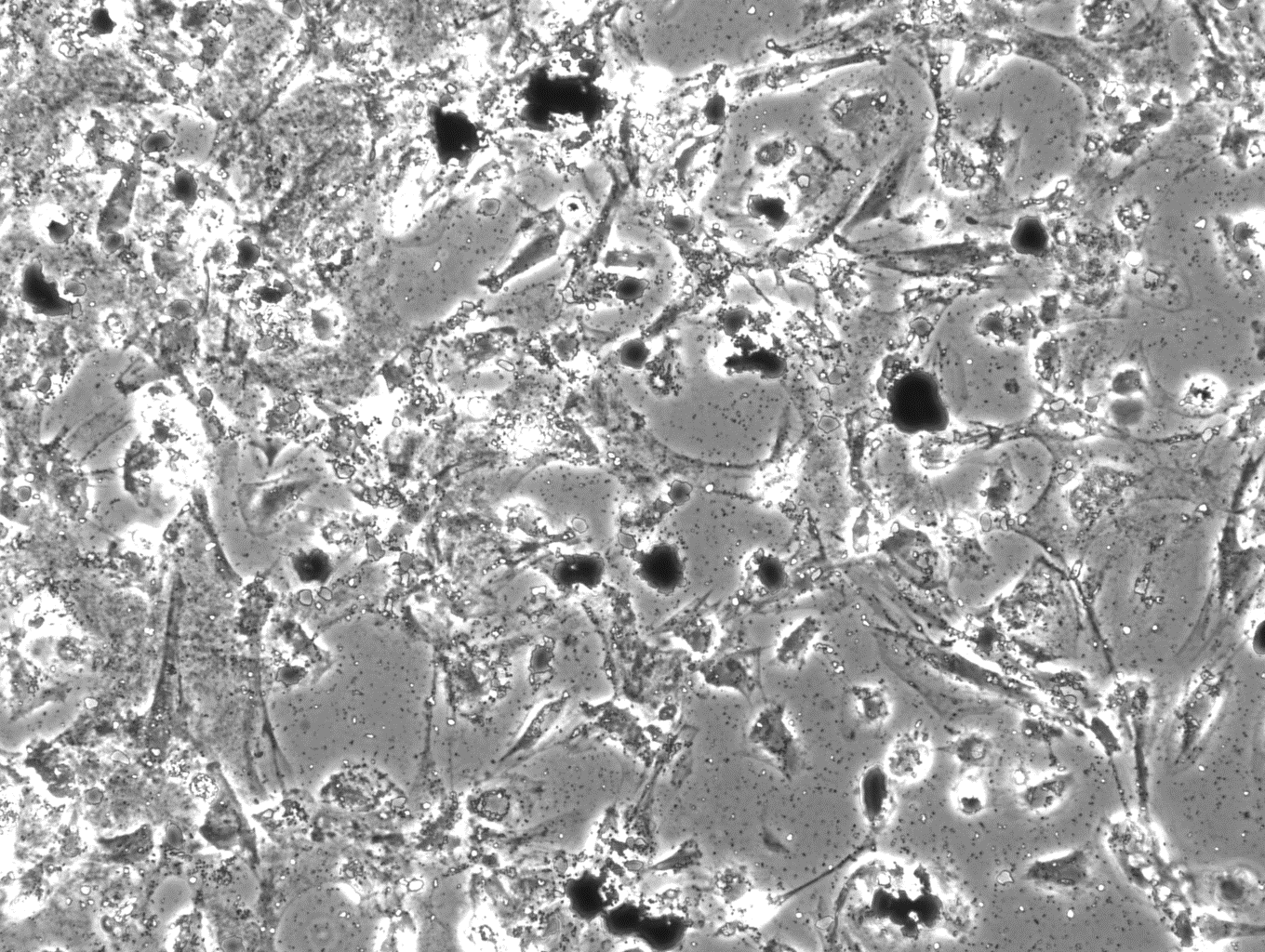 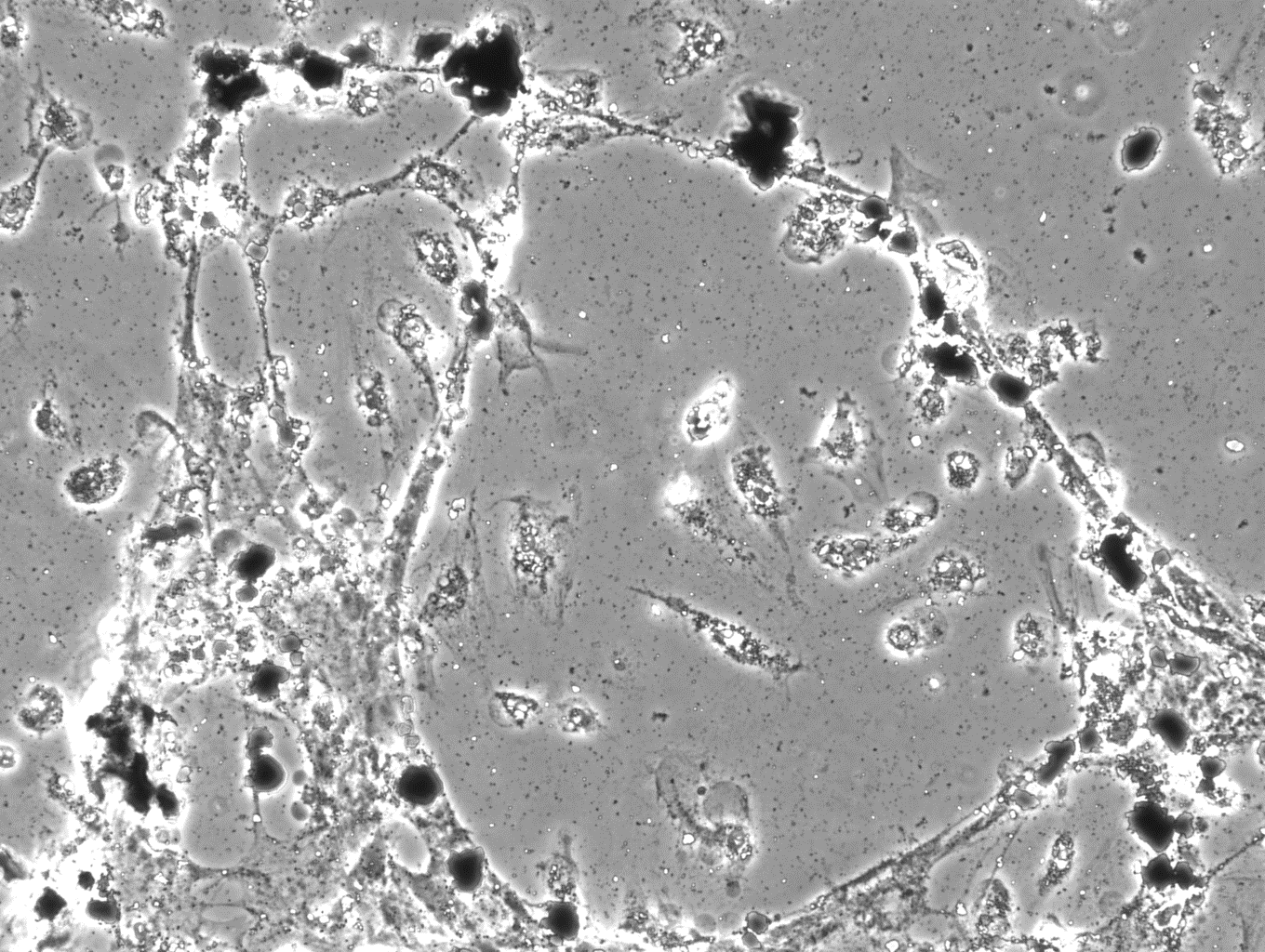 |
| 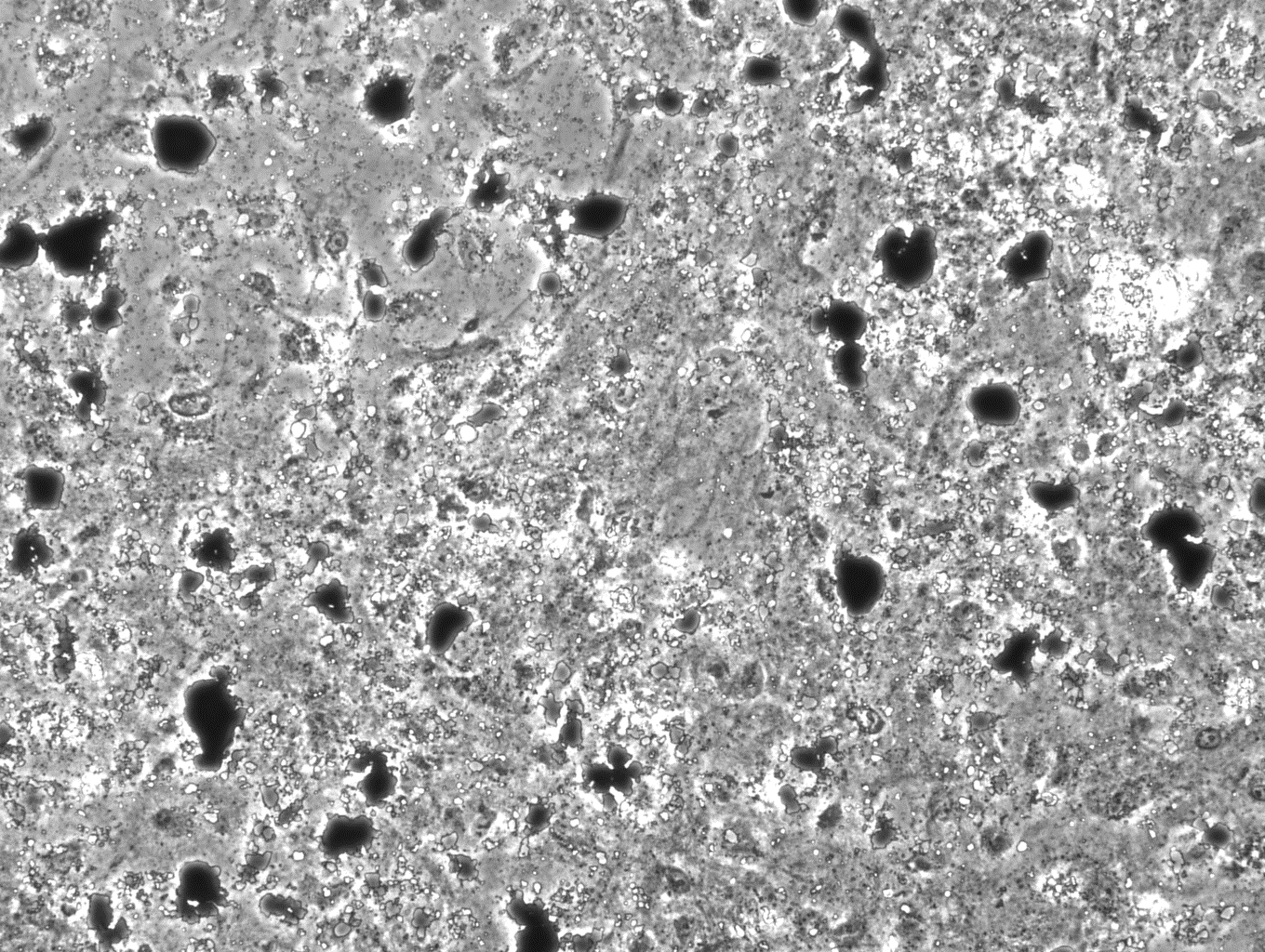 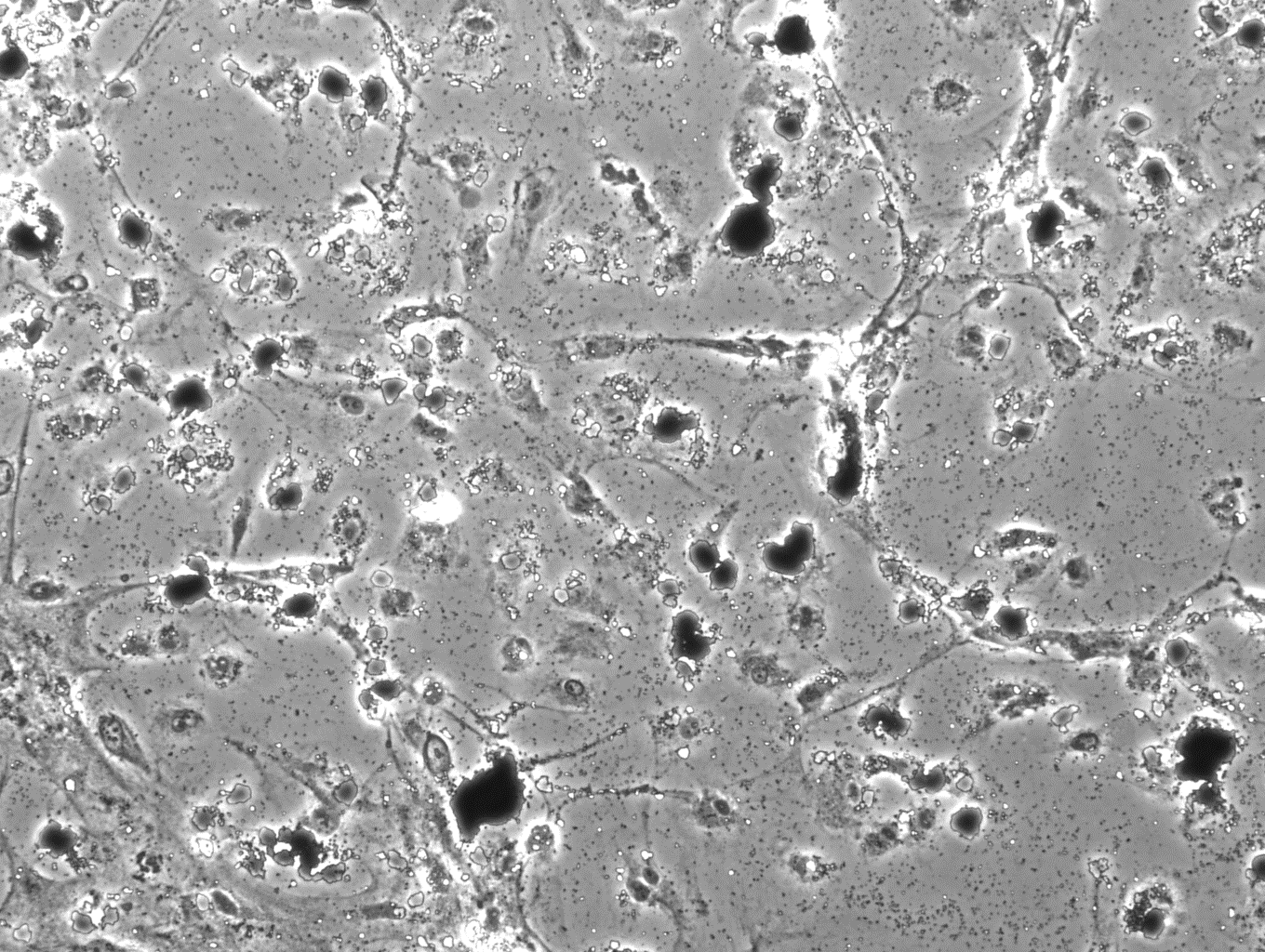 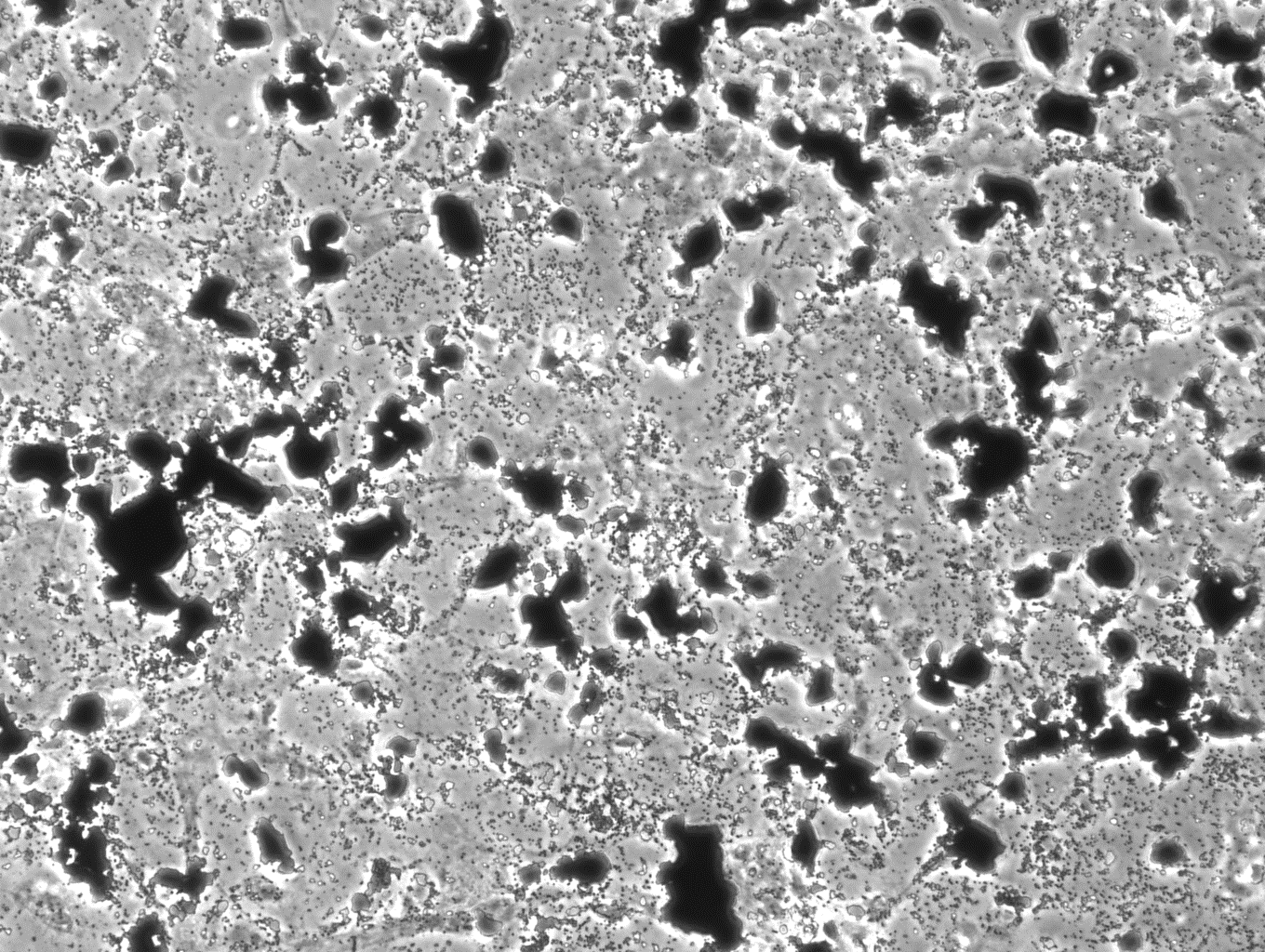 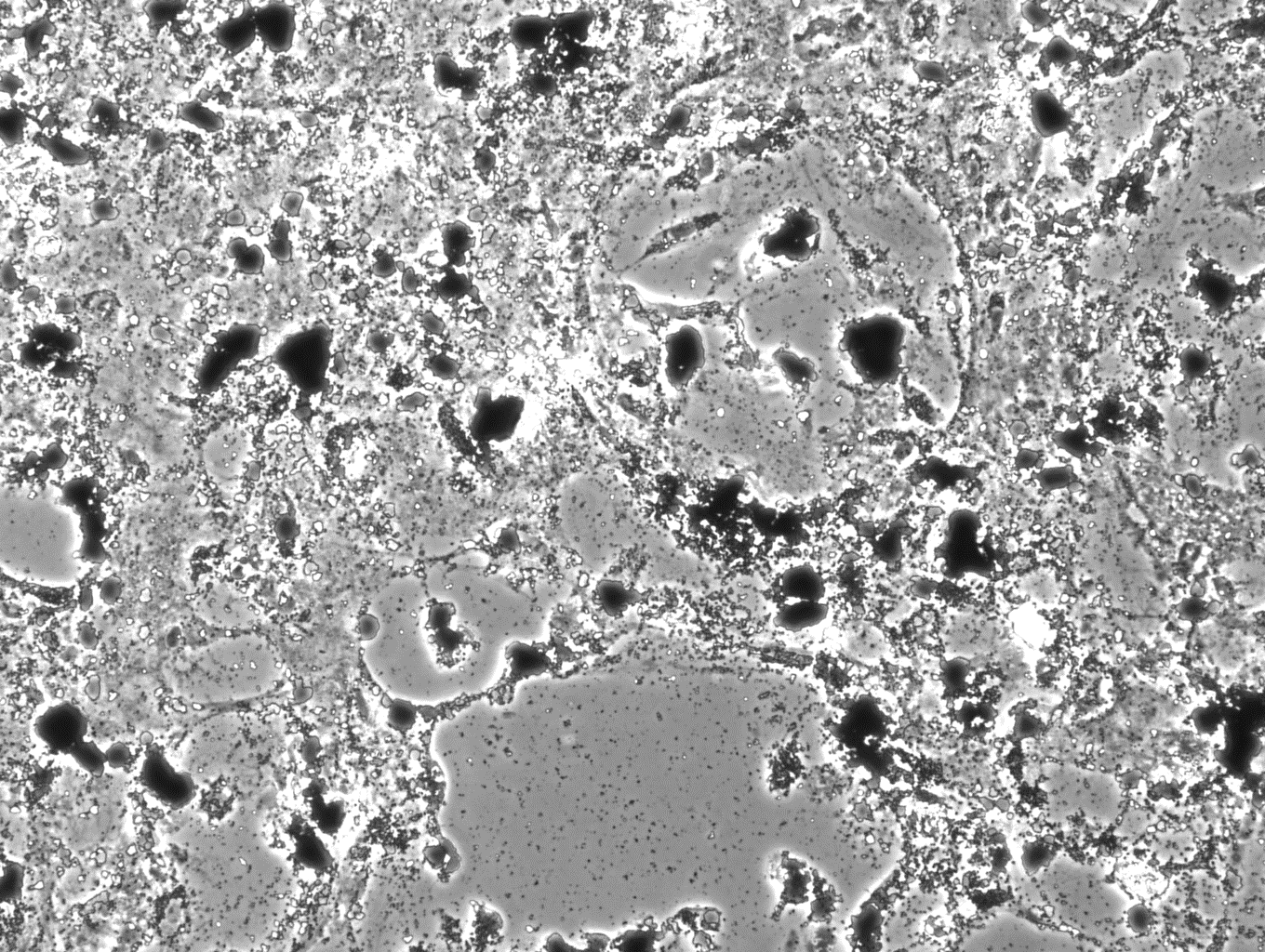 |

**Figure S5-1.** Bright-field image panel arrangements correspond to fluorescent images of Fig. 1. Each bright-field image in this panel shows the same cell sample’s location as corresponding fluorescent image of Fig. 1. The black spots or splotches that are DEPs can be clearly seen on these images.
