# Supplementary material for: Biophysical Assessment of Single Cell Cytotoxicity: Diesel Exhaust Particle-Treated Human Aortic Endothelial Cells
Source: PLoS One. 2012 May 25;7(5):e36885. doi: 10.1371/journal.pone.0036885 (PMC3360744; doi:10.1371/journal.pone.0036885)
Supplement: Information S6 — ROS generation measured by flow cytometry. (DOC) [file pone.0036885.s006.doc]

**Figure S6-1.** ROS generation induced by **10 µg/ml DEP** treatment quantitatively assessed by flow cytometry. Fluorescence intensity of control group (0 hour) was firstly normalized as 1, and those of experimental groups are presented as a relative ROS generation over control group. Results represent mean value of three separate experiments, and error bars are standard error of the mean. Analyses indicated that this low concentration of DEP introduction moderately elevated ROS formation in all four treatment groups. To analyze ROS generation at 10 µg/ml of DEPs, the dye 5-(and-6)-chloromethyl-2’,7’-dichlorodihydrofluorescein diacetate acetyl ester (CM-H_2_DCFDA, Ex/Em: 492-495/517-527 nm) (Invitrogen) was used to perform detection. Cells were stained according to the Invitrogen protocols. Briefly, the dye was first dissolved in DMSO and then diluted in PBS to a working concentration of 10 µM immediately before use. DEP-treated cells were harvested from growth media via centrifugation, and resuspended in the dye-PBS solution. Cells were then incubated at 37^o^C for 30 min, then washed to remove the loading dye-PBS solution, and returned to growth media and incubated at 37^o^C with 5% CO_2_ for 30 min. Cell samples were then assessed by flow cytometry.
